# Integrated microRNA, gene expression and transcription factors signature in papillary thyroid cancer with lymph node metastasis

**DOI:** 10.7717/peerj.2119

**Published:** 2016-06-15

**Authors:** Nurul-Syakima Ab Mutalib, Sri Noraima Othman, Azliana Mohamad Yusof, Shahrun Niza Abdullah Suhaimi, Rohaizak Muhammad, Rahman Jamal

**Affiliations:** 1 UKM Medical Molecular Biology Institute, Universiti Kebangsaan Malaysia, Cheras, Kuala Lumpur, Malaysia; 2 Department of Surgery, Faculty of Medicine, Universiti Kebangsaan Malaysia, Cheras, Kuala Lumpur, Malaysia

**Keywords:** Papillary thyroid carcinoma, Lymph node, MicroRNA, Gene expression

## Abstract

**Background**. Papillary thyroid carcinoma (PTC) is the commonest thyroid malignancy originating from the follicle cells in the thyroid. Despite a good overall prognosis, certain high-risk cases as in those with lymph node metastasis (LNM) have progressive disease and poorer prognosis. MicroRNAs are a class of non-protein-coding, 19–24 nucleotides single-stranded RNAs which regulate gene expression and these molecules have been shown to play a role in LNM. The integrated analysis of miRNAs and gene expression profiles together with transcription factors (TFs) has been shown to improve the identification of functional miRNA-target gene-TF relationships, providing a more complete view of molecular events underlying metastasis process.

**Objectives**. We reanalyzed The Cancer Genome Atlas (TCGA) datasets on PTC to identify differentially expressed miRNAs/genes in PTC patients with LNM-positive (LNM-P) versus lymph node negative (LNN) PTC patients and to investigate the miRNA-gene-TF regulatory circuit that regulate LNM in PTC.

**Results**. PTC patients with LNM (PTC LNM-P) have a significantly shorter disease-free survival rate compared to PTC patients without LNM (PTC LNN) (Log-rank Mantel Cox test, *p* = 0.0049). We identified 181 significantly differentially expressed miRNAs in PTC LNM-P versus PTC LNN; 110 were upregulated and 71 were downregulated. The five topmost deregulated miRNAs were hsa-miR-146b, hsa-miR-375, hsa-miR-31, hsa-miR-7-2 and hsa-miR-204. In addition, 395 miRNAs were differentially expressed between PTC LNM-P and normal thyroid while 400 miRNAs were differentially expressed between PTC LNN and normal thyroid. We found four significant enrichment pathways potentially involved in metastasis to the lymph nodes, namely oxidative phosphorylation (OxPhos), cell adhesion molecules (CAMs), leukocyte transendothelial migration and cytokine–cytokine receptor interaction. OxPhos was the most significantly perturbed pathway (*p* = 4.70E−06) involving downregulation of 90 OxPhos-related genes. Significant interaction of hsa-miR-301b with HLF, HIF and REL/NFkB transcription factors were identified exclusively in PTC LNM-P versus PTC LNN.

**Conclusion**. We found evidence of five miRNAs differentially expressed in PTC LNM-P. Alteration in OxPhos pathway could be the central event in metastasis to the lymph node in PTC. We postulate that hsa-miR-301b might be involved in regulating LNM in PTC via interactions with HLF, HIF and REL/NFkB. To the best of our knowledge, the roles of these TFs have been studied in PTC but the precise role of this miRNA with these TFs in LNM in PTC has not been investigated.

## Introduction

Papillary thyroid carcinoma (PTC) is the most common malignancy originating from the thyroid. Although the prognosis of PTC is generally good with a high 5-year survival rate, cases demonstrating certain clinicopathological parameters are progressive, have poorer prognosis and are considered as high-risk (Ito et al., 2009). Numerous classification systems for thyroid carcinoma have been established in order to classify high-risk cases such as AMES (Cady & Rosai, 1988), AGES (Hay et al., 1987), MACIS (Hay et al., 1993) as well as TNM (Sobin & Wittekind, 2002; AJCC, 2010). The TNM classification is the most recent classification system and is based on size and extrathyroid extension (T), lymph node involvement (N), distant metastasis (M) and patient’s age.

MicroRNAs (miRNAs), firstly identified in *Caenorhabditis elegans*, are a class of endogenous (non-protein-coding), 19–24 nucleotides single-stranded RNAs that derive from a stem-loop precursor to inhibit gene expression by binding primarily to the 3′-UTR of specific ‘target’ messenger RNA (mRNAs). MiRNAs that bind with perfect or nearly perfect complementarity to protein-coding mRNA sequences induce the RNA-mediated interference (RNAi) pathway, resulting in the disruption of mRNA stability and/or translation (Bartel, 2009). Dysregulation of miRNAs expression in human cancers have been demonstrated by many studies (Iorio & Croce, 2012). Through expression profiling studies, miRNAs were shown to be linked to tumor development, tumor progression, and response to treatment, signifying their potential use as biomarkers for diagnosis and prognosis (Iorio & Croce, 2012). MiRNAs have also been shown function as biomarkers in predicting lymph node metastasis (LNM). There was a positive correlation between high hsa-miR-21 expression with tumor stage and LNM in patients with breast cancer (Yan et al., 2008), and the development of distant metastases in colorectal cancer patients (Slaby et al., 2007). Most recently, hsa-miR-1207-5p was suggested as a useful biomarker in the prediction of LNM in gastric cancer (Huang et al., 2015) and head and neck cancer (De Carvalho et al., 2015).

The current approach of miRNA target gene prediction via *in silico* analysis is built upon sequence similarity search and thermodynamic stability (Alexiou et al., 2009). Nevertheless, it is acknowledged that the results of *in silico* target prediction algorithms suffer from very low specificity (Alexiou et al., 2009). The combination of *in silico* target predictions with miRNA and gene expression profiles has been proven to improve the identification of functional miRNA-target gene relationships (Nunez-Iglesias et al., 2010; Ma et al., 2011). As miRNAs act prevalently through degradation of the target genes, expression profiles of miRNA and target genes/transcripts are predicted to be inversely correlated (Bisognin et al., 2012). Another regulatory component, the transcription factors (TF), has also been shown to activate or repress miRNA expression level, further adding to the complexity of gene regulation. Efforts have been made to comprehend the mechanism of miRNAs in decreasing target genes expression; however the study of miRNA regulation by TFs (TF–miRNA regulation) is rather limited (Wang et al., 2010a).

The Cancer Genome Atlas (TCGA) Research Network recently published a molecular characterization of 507 PTCs and 59 matched normal adjacent tissues with respect to genomic, transcriptomic and proteomic signatures together with DNA methylation profiles, clinical and pathological features (Cancer Genome Atlas Research Network, 2014). Data were collected through several studies across different institutions, thus creating a comprehensive dataset of PTC samples. Through unsupervised clustering methods, TCGA yielded six subtypes for miRNA expression and five for gene expression. However, miRNA and gene expression profiles between PTC with and without LNM were not comprehensively discussed. Here we reanalyzed these TCGA datasets on PTC with the aim of identifying differentially expressed miRNAs/genes in PTC patients with LNM-positive (LNM-P) as compared to lymph node negative (LNN) PTC patients and to investigate the miRNA-gene-TF regulatory circuit that governs LNM in PTC.

## Materials and Methods

### TCGA papillary thyroid cancer dataset

We used the TCGA-generated microRNA sequencing (miRNAseq) and mRNAseq data for 495 tumors and 59 normal thyroid samples (Cancer Genome Atlas Research Network, 2014). Metadata containing clinical information including *BRAF* V600E mutation status was obtained from cBioPortal (http://www.cbioportal.org/study.do?cancer_study_id=thca_tcga_pub#clinical) while miRNAseq and mRNAseq of 507 PTC patients were obtained from the TCGA Data Portal (https://tcga-data.nci.nih.gov/tcga/dataAccessMatrix.htm) (accessed from March 27, 2015 to May 25, 2015). Information were available for 507 PTC patients. The list of patients from the metadata was then filtered for PTC patients with N0, N1, N1a, and N1b, resulting in a total of 421 PTC patients out of the 507 patients (86 patients were excluded due to unavailability of node status). The clinical parameters are presented in Table 1.

**Table 1:** Patient characteristics and integrated profiles in the TCGA PTC cohort.

Variables	PTC LNN	PTC LNM-P		
	N0 (*n* = 213)	N1 (*n* = 53)	N1a (*n* = 86)	N1b (*n* = 66)
Age range (years)	15–85	19–83	18–83	19–89
Mean age	49.4	41.9	43.5	48.4
Gender (*n*)				
Male	50 (23.5%)	14 (26.4%)	25 (29.1%)	27 (40.9%)
Female	163 (76.5%)	39 (73.6%)	61 (70.9%)	39 (59.1%)
Disease free status				
Recurred/progressed	5 (2.3%)	7 (13.2%)	6 (7%)	6 (9.1%)
Disease free	178 (83.6%)	41 (77.4%)	75 (87.2%)	47 (71.2%)
Unknown	30 (14.1%)	5 (9.4%)	5 (5.8%)	13 (19.7%)
Disease free (range in months)	0.03–155	0–131	0–157	0.2 –46
Mean disease-free survival	23.6 (*n* = 183)	34.5 (*n* = 48)	21.5 (*n* = 81)	13.5 (*n* = 53)
Overall survival status				
Deceased	35 (16.4%)	12 (22.6%)	11 (12.8%)	19 (28.8%)
Alive	178 (83.6%)	41 (77.6%)	75 (87.2%)	47 (71.2%)
Overall survival (range in months)	0.03–155	0–131	0–157	0.2 –97.7
Mean overall survival	24.3 (*n* = 182)	35.2 (*n* = 43)	21.4 (*n* = 75)	15.2 (*n* = 50)
Extrathyroidal extension				
None	160 (75.1%)	31 (58.5%)	49 (57%)	37 (56.1%)
Minimal (T3)	42 (19.7%)	14 (26.4%)	33 (38.4%)	23 (34.8%)
Moderate/advanced (T4a)	3 (1.4%)	5 (9.4%)	1 (1.2%)	4 (6.1%)
Very advanced (T4b)	0 (0%)	1 (1.9%)	0 (0%)	0 (0%)
Unknown	8 (3.8%)	2 (3.8%)	3 (3.5%)	2 (3%)
BRAF status				
Mutated	94 (44.1%)	25 (47.2%)	53 (61.6%)	32 (48.9%)
Wild type	119 (55.9%)	28 (52.8%)	33 (38.4%)	34 (51.5%)

Only samples with paired miRNAseq and mRNAseq data were selected, resulting in exclusion of additional three patients. In the end, we obtained a total of 418 patients’ dataset which includes 213 patients with PTC LNN (N0) and 205 PTC LNM-P (53 patients with N1, 86 patients with N1a, 66 patients with N1b) (Table S1). Combined with 59 normal thyroid tissues, the total of datasets included in this study were 477. The miRNA and gene expression datasets consisting of 1,046 human miRNAs and 20,531 genes, respectively, were used for subsequent analysis.

### Survival analyses

Kaplan–Meier survival analysis was carried out on disease-free and overall survival duration of TCGA PTC patients for whom follow-up details were available. Overall survival is defined as the duration from the date of diagnosis to death (due to all causes) while disease-free survival is defined as the duration from the date of the diagnosis to the date of recurrence, second cancer, or death due to all causes (whichever occurred first) (Schvartz et al., 2012). Curves were compared by univariate (log-rank) analysis. Statistical analyses were performed using GraphPad Prism version 6 (GraphPad, San Diego, CA, USA). *P* values ≤0.05 were considered significant.

### Clinical specimen and total RNA isolation

Ten fresh frozen tumour-adjacent normal PTC tissues specimens from UKMMC-UMBI Biobank were subjected to cryosectioning and Haematoxylin and Eosin (H&E) staining. This part of research was approved by the Universiti Kebangsaan Malaysia Research Ethics Committee (UKMREC) (reference: UKM 1.5.3.5/244/UMBI-2015-002). A written informed consent had been signed by these 10 subjects included in validation phase according to institution’s rules and regulations. All the slides were reviewed by the pathologist to assess the percentage of tumour cells and normal cells. Only tumour tissues which contain >80% cancer cells and normal tissues with <20% necrosis were subjected to nucleic acid extraction. Total RNA including miRNA was isolated from the frozen samples using AllPrep DNA/RNA/miRNA Isolation Kit (Qiagen, Hilden, Germany) according to the manufacturer’s protocol. The total RNA quality and quantity were assessed via absorbance spectrophotometry on a Nanodrop 1000 instrument (Thermo Scientific, Wilmington, DE, USA) and Qubit™ fluorometer (Invitrogen, USA). Integrity of RNA was assessed using Eukaryote Total RNA Nano chip on Bioanalyzer 2100 (Agilent Technologies, Santa Clara, USA). Only total RNA with RNA Integrity Number (RIN) of at least 6 were used for subsequent steps. Eukaryote Small RNA chip (Agilent Technologies, Santa Clara, USA) was used for determination of concentration and percentage of small RNA.

### Library preparation and next generation sequencing

MiRNA libraries were prepared using Illumina Truseq Small RNA library preparation kit (Illumina, SanDiego, USA) following manufacturer’s protocol. Briefly, 3′ and 5′ adapters were sequentially ligated to the ends of small RNAs fractionated from 1 µg of total RNA, and reverse transcribed to generate cDNA. The cDNA was amplified using a common primer complementary to the 3′ adapter, and a primer containing 1 of 48 index sequences. Samples were size-selected (145–160 bp fragments) on a 6% polyacrylamide gel, purified, quantified and pooled for multiplexed sequencing. The resulting pooled libraries were normalized to 2 nM and were hybridized to oligonucleotide-coated single-read flow cells for cluster generation using HiSeq^®^ Rapid SR Cluster Kit v2 on Hiseq 2500. Subsequently the clustered pooled microRNA libraries were sequenced on the HiSeq 2500 for 50 sequencing cycles using HiSeq^®^ Rapid SBS Kit v2 (50 Cycle). Base calling was performed using CASAVA (v.1.8.2) (Illumina, San Diego, CA, USA) and short-read sequences in FASTQ format were used for downstream analysis.

### Bioinformatics analyses

The miRNASeq and RNASeq V2 level 3 data from TCGA were used exclusively. The normalised expression (reads per million or RPM) of all miRNAs was log_2_-transformed and used for fold change calculation. The RNAseq by Expectation-Maximization (RSEM) values (from files with the extension .rsem.genes.results) were used to quantify messenger RNA (mRNA) expression levels. The RSEM algorithm is a statistical model that estimates RNA expression levels from RNA sequencing counts (Li & Dewey, 2011). We then performed the Students’ unpaired *t*-test with a Benjamini Hochberg false discovery rate (FDR) multiple testing correction and log_2_ fold change calculation using Bioconductor version 3.1 (BiocInstaller 1.18.2) (Gentleman et al., 2004) in R version 3.2.0 (R Development Core Team, 2008) (Files S1 and S2). Downregulated genes will have negative log_2_ values while upregulated genes will have positive log_2_ values. Statistical significance is denoted as *p* ≤ 0.05. Heatmaps were created using GeneE from the Broad Institute (http://www.broadinstitute.org/cancer/software/GENE-E) while Venn diagrams were created using Venn online tool (http://bioinformatics.psb.ugent.be/webtools/Venn). All other figures were created or labelled using Adobe Photoshop.

Analysis of the miRNAseq data from our in house experiment were performed using BaseSpace miRNA Analysis app version 1.0.0 (Illumina, San Diego, CA, USA) using the default setting. Briefly, adapters were trimmed using cutadapt, the trimmed reads were mapped on miRNA precursors using SHRiMPS aligner, the reads associated to mature miRNAs were counted and differential expression between experimental conditions were analysed using DESeq2 (Cordero et al., 2012). The expression, log_2_ fold change and adjusted *p*-value of hsa-miR-146b, hsa-miR-375, hsa-miR-31, hsa-miR-7-2 and hsa-miR-204 were then extracted from the overall results.

### Pathway enrichment analysis and integrated analysis of miRNA and gene expression

The functions and pathways of the differentially expressed genes were annotated and analysed using the annotation tools from the Database for Annotation, Visualization and Integrated Discovery (DAVID) (Huang, Sherman & Lempicki, 2009a; Huang, Sherman & Lempicki, 2009b) according to the steps described in these publications. The identified genes were also jointly annotated against the Kyoto Encyclopedia of Genes and Genomes (KEGG) database (Kanehisa & Goto, 2000). The genes that were annotated in the KEGG database as being involved in signaling pathways were subjected to further analysis. Pathways with Benjamini-adjusted *p* value ≤0.05 were considered to be statistically significant.

Integration of the miRNAs dataset with gene expression dataset and calculation of correlation were performed in MAGIA2, a web tool for the integrated analysis of target predictions, miRNA and gene expression data (Bisognin et al., 2012). MiRNA target predictions include transcription factor binding sites (TFBS) within miRNA and gene promoters. In this analysis, matched expression data matrices of significantly dysregulated miRNAs and genes (BH adjusted *p* value ≤0.05) were uploaded for integrated analysis. EntrezGene IDs and DIANA-microT (Maragkakis et al., 2009) target prediction algorithms were selected. Anticorrelated expressions were investigated between miRNA and their putative target genes using Pearson correlation measure.

## Results

### The effect of lymph node status on survival duration of TCGA PTC patients

**Figure 1:**
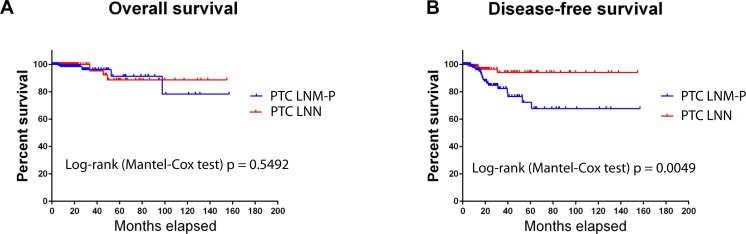
Survival analysis of PTC with LNM and PTC without LNM.

Overall survival in PTC patients was not influenced by LNM status (Fig. 1A); however, PTC patients with LNM has significantly shorter disease-free survival rate compared to PTC patients without LNM (Log-rank Mantel Cox test, *p* = 0.0049; Fig. 1B).

### Differentially expressed miRNAs

**Figure 2:**
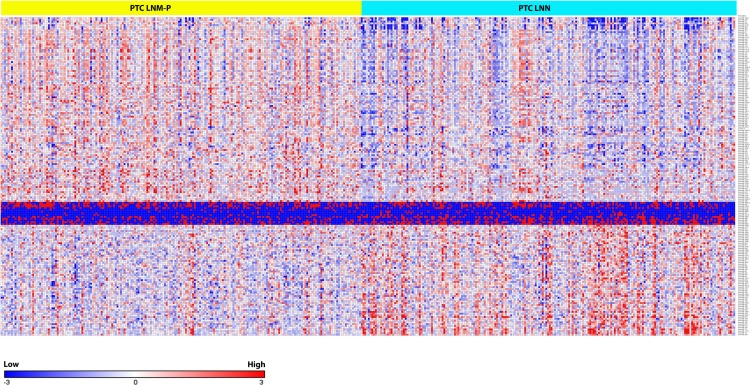
Heat map of the 181 differentially expressed miRNAs in PTC LNM-P and LNN (Student’s *T*-test with BH corrected *p* value ≤ 0.05).

**Figure 3:**
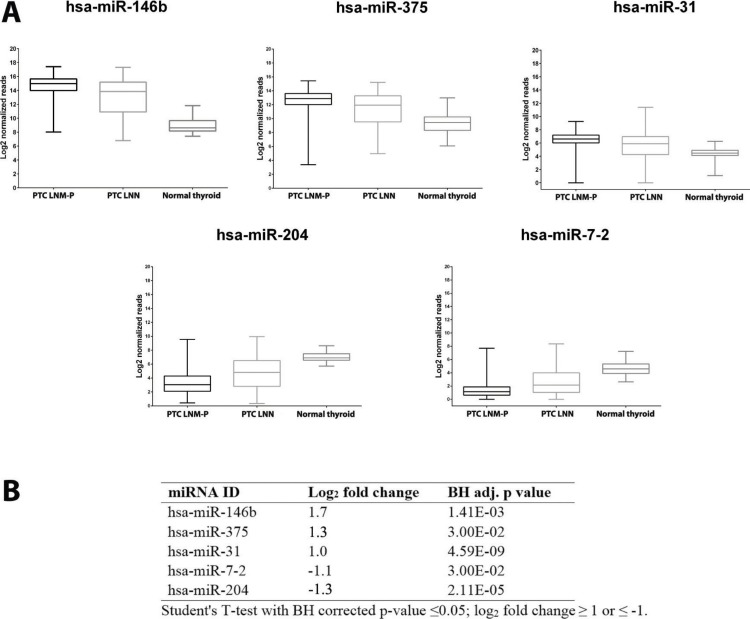
Expression levels of five selected miRNAs deregulated in PTC. Boxplots (A) illustrate log_2_ normalized miRNA reads in PTC LNM-P, PTC LNN and normal thyroid. Table (B) showing log2 fold change and *p* value of selected miRNAs in PTC LNM-P compared to PTC LNN.

We identified 181 miRNAs which were significantly differentially expressed in PTC LNM-P versus PTC LNN (BH corrected *p* value ≤0.05). Among the 181 miRNAs significantly expressed in PTC LNM-P versus PTC LNN, 110 were upregulated and 71 were downregulated (Table S2). Figure2 illustrates a heatmap representing the expression levels of 181 deregulated miRNAs in PTC LNM-P versus PTC LNN. The list of top deregulated miRNAs includes hsa-miR-146b, hsa-miR-375, hsa-miR-31, hsa-miR-7-2 and hsa-miR-204 (log_2_ fold change 1.7, 1.3, 1, −1.1 and −1.3, respectively, Fig. 3). On the other hand, 395 miRNAs were differentially expressed between PTC LNM-P and normal thyroid while 400 miRNAs were differentially expressed between PTC LNN and normal thyroid (Figs. S1 and S2, respectively). The list of miRNAs significantly deregulated in PTC LNM-P and PTC LNN compared to normal thyroid is included in Table S3.

We then determine the expression of these top deregulated miRNAs in a small set of validation experiment consisted of five pairs of tumour-adjacent normal from each PTC LNM-P and PTC LNN cases (total of 20 samples comprised of five PTC LNM-P, five PTC LNN and 10 adjacent normal thyroid tissues from each patient). As illustrated in Fig. 3B, hsa-miR-146b was significantly upregulated in PTC LNM-P versus adjacent normal thyroid (log_2_ fold change 6.0) and in PTC LNN versus adjacent normal thyroid (log_2_ fold change 4.7). Similar trends were observed for hsa-miR-375, hsa-miR-31 and hsa-miR-204 in PTC LNM-P versus adjacent normal thyroid (log_2_ fold change 3.6, 3.1 and −3.4, respectively, Fig. 3B). However, downregulation of hsa-miR-7-2 in PTC LNM-P versus adjacent normal thyroid did not reach statistical significance. On the other hand, expression of hsa-miR-375, hsa-miR-7-2 and hsa-miR-204 in PTC LNN versus adjacent normal thyroid were in concordance with our analysis using the TCGA data (log_2_ fold change 3.6, −2.3 and −2.7, Fig. 3B).

These findings did not deviate much from our analysis using the TCGA PTC data with the exception to hsa-miR-7-2 in PTC LNM-P versus adjacent normal thyroid and hsa-miR-31 in PTC LNN versus adjacent normal thyroid which failed to reach statistical significance (Table S2). This could be explained due to the fact that our validation samples were tumour-adjacent normal tissues while TCGA PTC specimens were of unpaired normal tissues. However, the differential expression of these five top deregulated miRNAs did not reach statistical significant when we compared between PTC LNM-P and PTC LNN (Fig. 3B). This might be due to small sample size in our validation study.

### Differentially expressed genes

Initial filtering revealed 8,611 significantly deregulated genes in PTC LNM-P versus PTC LNN, 14,192 genes in PTC LNM-P versus normal thyroid and 13,392 genes in PTC LNN versus normal thyroid. There were 4,135 upregulated and 4,476 downregulated genes in PTC LNM-P relative to PTC LNN. By increasing the stringency of selection to genes with log_2_ fold change ≥1 or ≤ − 1, 407 genes were identified as strongly deregulated. Among the strongly deregulated genes were *SFTPB*, *CLDN10*, *DIO1* and *MT1G* (log_2_ fold change 3.1, 2.9, −2.2 and −2.5 respectively, Table S4). Various cancer-related genes were also differentially expressed significantly, including *BRAF*, *BRCA2*, *VEGFA*, *VEGFB*, *RET*, *PIK3CA*, *CTNNB1* and *GNAS* (Table S4).

### Enriched pathways in PTC LNM-P

**Figure 4:**
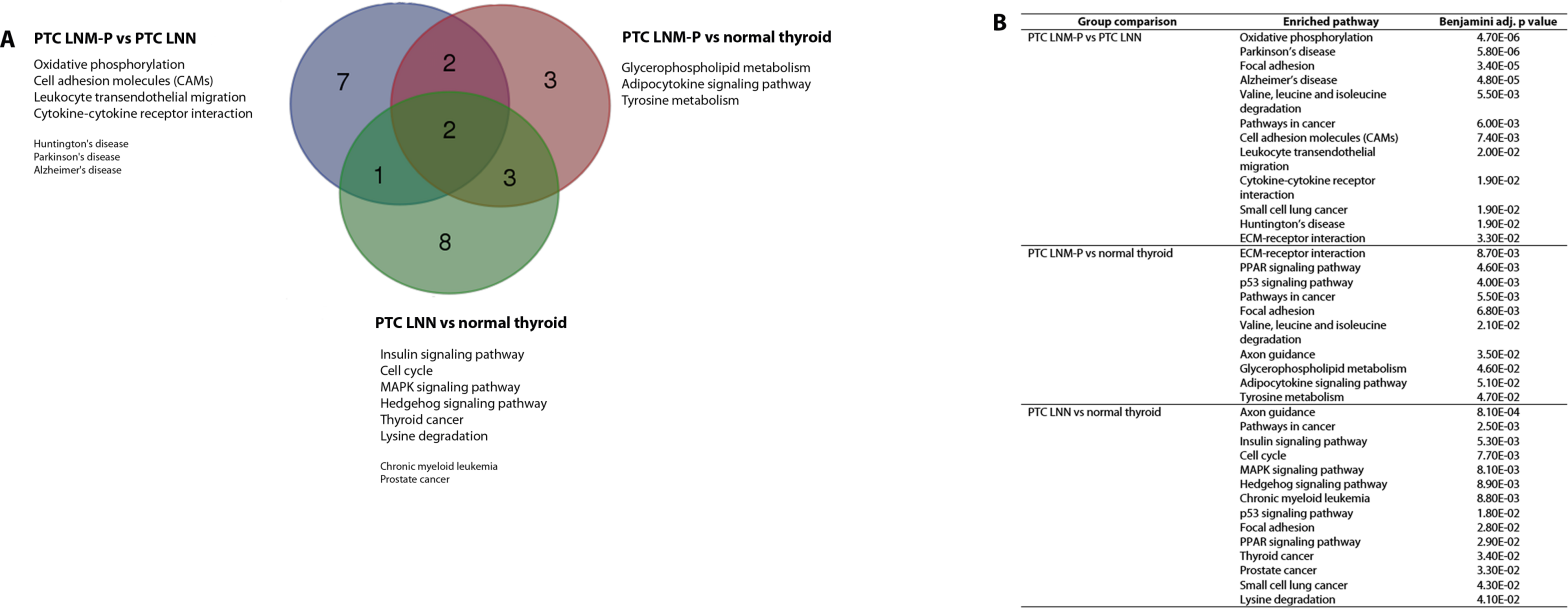
Significantly enriched pathways in PTCs. Significant KEGG pathway associations to 8611 significantly deregulated genes in PTC LNM-P versus PTC LNN, 14,192 genes in PTC LNM-P versus normal thyroid and 13,392 genes in PTC LNN versus normal thyroid.

**Figure 5:**
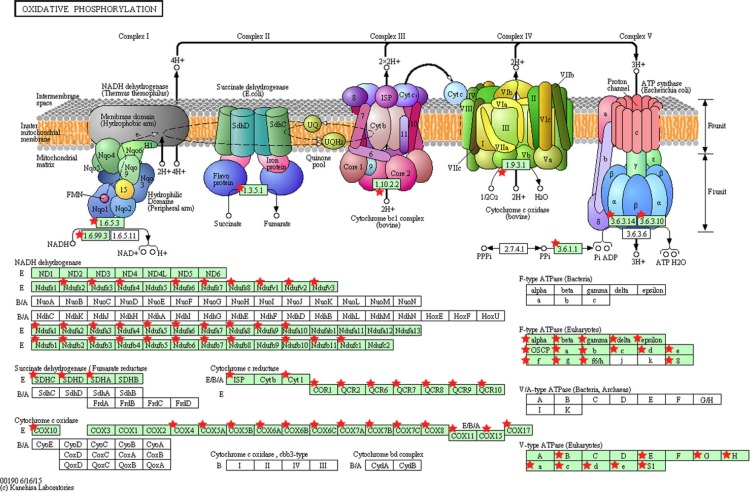
KEGG pathway map illustrating oxidative phosphorylation in human. The OxPhos-related genes significantly altered in PTC LNM-P compared to PTC LNN were depicted with red star. Pathway figure was obtained from KEGG (Kyoto Encyclopedia of Genes and Genomes) (Kanehisa & Goto, 2000; Kanehisa et al., 2016) in DAVID analysis.

The significantly dysregulated genes in PTC LNM-P versus PTC LNN were mainly enriched in 12 KEGG pathways including oxidative phosphorylation (OxPhos), Parkinson’s disease, focal adhesion, Alzheimer’s disease, valine, leucine and isoleucine degradation, pathways in cancer, cell adhesion molecules (CAMs), leukocyte transendothelial migration, cytokine–cytokine receptor interaction, small cell lung cancer, Huntington’s disease and extracellular matrix receptor interaction (Fig. 4A). When we overlapped the results from the three comparison groups (PTC LNM-P versus PTC LNN, PTC LNM-P versus normal thyroid and PTC LNN versus normal thyroid), four unique pathways potentially involved in metastasis to the lymph nodes were significantly enriched, namely, oxidative phosphorylation (OxPhos), cell adhesion molecules (CAMs), leukocyte transendothelial migration and cytokine–cytokine receptor interaction pathways (Fig. 4A). The oxidative phosphorylation pathway was the most significantly perturbed (*p* = 4.70E−06) with general downregulation of 90 OxPhos-related genes (Fig. 5). Focal adhesion and pathways in cancer were commonly enriched in all the three group comparisons. Pathways in cancer is a collection of general cancer-related pathways and is an indication that many essential carcinogenic processes may be under the influence of dysregulated miRNAs (Pizzini et al., 2013). On the other hand, ECM-receptor interaction pathway and the valine, leucine and isoleucine degradation pathway were commonly enriched only in PTC LNM-P versus PTC LNN and PTC LNM-P versus normal thyroid but were not enriched in PTC LNN versus normal thyroid (Fig. 4B).

### Integrated mixed regulatory circuits, involving miRNAs, genes and TFs in PTC LNM-P

**Figure 6:**
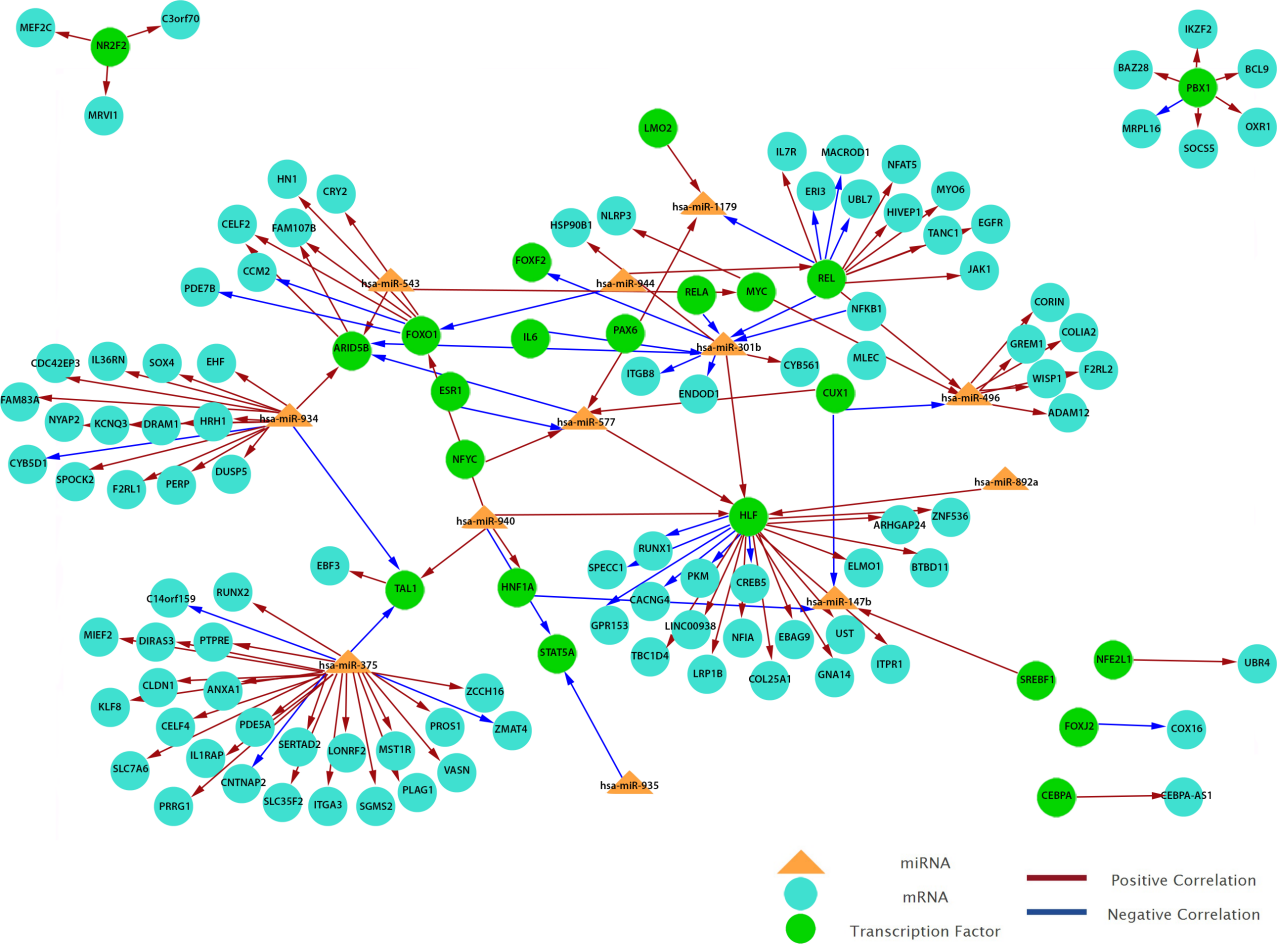
Grand view of top 200 regulatory circuits constructed using significantly dysregulated miRNAs and genes in PTC LNM-P compared to PTC LNN.

To obtain a more comprehensive insight into the molecular circuits behind LNM in PTC, we focused on functional miRNA-target relationships by performing an *in silico* integration between differentially expressed miRNAs and genes using MAGIA2. Transcription factors-miRNA (TF-miRNA) prediction was based on mirGen2.0 database (Friard et al., 2010) and TransmiR (Wang et al., 2010a), whereas the TF–gene interactions were acquired from the ‘TFBS conserved’ track of the University of California Santa Cruz (UCSC) genome annotation for humans (version hg19) (Bisognin et al., 2012). Our results show that 12 miRNAs are involved in the strongest 200 interactions and they were identified as significant by MAGIA2. Hsa-miR-147b, hsa-miR-301b, hsa-miR-375, hsa-miR-496, hsa-miR-543, hsa-miR-577, hsa-miR-765, hsa-miR-892a, hsa-miR-934, hsa-miR-935, hsa-miR-940 and hsa-miR-944 were predicted to activate or inhibit 3,746 genes and 1,987 TFs (Fig. 6). Hsa-miR-577 and hsa-miR-147b consistently appeared in the top 20 regulatory circuits across all group comparisons. Interestingly, hsa-miR-301b appeared in both of the top 20 circuits in PTC LNM-P versus PTC LNN or normal thyroid but was absent in PTC LNN versus normal thyroid (Fig. S3).

## Discussion

In this study, we explored the landscape of miRNA and mRNA expression of PTC using data obtained from the TCGA THCA project aiming to identify key pathways involved in lymph node metastasis. Our analysis revealed 110 upregulated miRNAs and 71 downregulated miRNAs in PTC LNM-P versus PTC LNN. The top deregulated miRNAs includes hsa-miR-146b, hsa-miR-375, hsa-miR-31, hsa-miR-7-2 and hsa-miR-204. Our findings are supported by several other similar studies, and in particular hsa-miR-146b, which was reported to be upregulated in PTC LNM-P versus PTC LNN (Lee et al., 2013; Yang et al., 2013; Acibucu et al., 2014; Deng et al., 2015).

Hsa-miR-146 is one of the widely studied miRNAs in thyroid cancers and has been shown to be frequently upregulated in PTC (He et al., 2005; Pallante et al., 2006; Tetzlaff et al., 2007; Chen et al., 2008; Yip et al., 2011; Chou et al., 2010; Chou et al., 2013; Sun et al., 2013), anaplastic thyroid cancer (Fassina et al., 2014) and follicular thyroid cancer (FTC) (Wojtas et al., 2014). Functional analyses of hsa-miR-146 revealed its involvement in various cellular functions including migration, invasion, proliferation, colony-forming ability, cell cycle, and resistance to chemotherapy-induced apoptosis in *BRAF*-mutated cell lines (Chou et al., 2013; Deng et al., 2015; Geraldo, Yamashita & Kimura, 2012). Using multivariate logistic regression analysis, Chou and colleagues, (2013) demonstrated that increased hsa-miR-146b expression is one of the independent risk factors for poor prognosis in PTC, implicating the potential of this miRNA as a prognostic marker.

The genes targeted by hsa-miR-146b are mostly unknown, and to date there are only two genes which has been reported as the direct targets of this miRNA in PTC. Geraldo, Yamashita & Kimura (2012) reported *SMAD4*, an important member of the transforming growth factor *β* (TGF-*β*) signaling pathway, as the target of hsa-miR-146b-5p. The direct binding of hsa-miR-146b-5p on the *SMAD4* UTR was confirmed via a luciferase reporter assay and the inhibition of hsa-miR-146b-5p expression resulted in significantly increased SMAD4 gene and protein expression levels in the human PTC cell lines. Furthermore, the inhibition of hsa-miR-146b-5p increased the cellular response to the TGF-*β* anti-proliferative signal, leading to significant reduction of cell proliferation (Geraldo, Yamashita & Kimura, 2012). In a more recent study, the Zinc Ring Finger 3 (ZNRF3) gene was revealed as a direct target of hsa-miR-146b-5p and this miRNA was shown to stimulate cell migration, invasion and epithelial-to-mesenchymal transition (EMT) by downregulating *ZNRF3* (Deng et al., 2015). Another study showed that *ZNRF3* inhibits Wnt signaling by interacting with FZD and LRP 5/6 complexes, hence promoting Wnt receptor ubiquitination and degradation (Hao et al., 2012). Hsa-miR-146b-5p increases the cell surface levels of FZD6 and LRP6 via suppression of *ZNRF3*, causing enhanced Wnt/*β*-catenin signaling. These findings revealed a novel mechanism of hsa-miR-146b-5p in mediating the induction of EMT and implied the role of *ZNRF3* as a tumor suppressor in PTC (Deng et al., 2015). Additional efforts to identify genes controlled by hsa-miR-146b associated with LNM will eventually revealed new biomarkers that can be utilized to correlate with disease outcome in PTC patients.

Hsa-miR-204 expression in PTC LNM-P is significantly lower than in PTC LNN. This is the first report showing the downregulation of hsa-miR-204 in PTC LNM-P. This miRNA was also downregulated in PTC compared to adjacent normal thyroid tissue and noncancerous thyroid (Swierniak et al., 2013). It is likely that hsa-miR-204 is downregulated in PTC compared to normal or benign thyroid disease and is further supressed when lymph node metastasis occurs. This miRNA is known as a tumor suppressor miRNA and is downregulated in various cancers including renal clear cell carcinoma (Gowrishankar et al., 2014), minimal deviation adenocarcinoma (MDA) of uterine cervix (Lee et al., 2014) and breast cancer (Li et al., 2014). This miRNA has also been shown to have a prognostic value; low level of hsa-miR-204-5p expression was correlated with LNM, advanced stage and low survival rate in endometrial cancer (Bao et al., 2013), and also poor prognosis in colorectal cancer (Yin et al., 2014). *In vitro* functional analyses revealed the involvement of hsa-miR-204 in inhibiting the clonogenic growth, migration and invasion of endometrial carcinoma cells (Bao et al., 2013). In addition, restoration of hsa-miR-204-5p expression supressed cell proliferation, migration, invasion and induced apoptosis and chemotherapeutic sensitivity in colorectal cancer cell (Yin et al., 2014).

The validated targets for hsa-miR-204 in PTC are also not well-characterized. To date there is only one study investigating the functional role of hsa-miR-204 in PTC (Liu et al., 2015). Enforced expression of hsa-miR-204-5p inhibited cell proliferation and induced apoptosis and cell cycle arrest in PTC cell lines (TCP-1 and BCPAP). In addition, hsa-miR-204-5p also inhibits PTC cell tumorigenicity *in vivo* (Liu et al., 2015). Bioinformatics prediction analyses using three algorithms (miRanda, Pictar, and TargetScan) revealed the insulin-like growth factor-binding protein 5 (IGFBP5), a gene playing an essential role in carcinogenesis (Beattie et al., 2006), as a potential target of hsa-miR-204-5p. Luciferase reporter assay confirmed the direct binding of hsa-miR-204-5p to the 3′ UTR of *IGFBP5* (Liu et al., 2015). In the same study, hsa-miR-204-5p and *IGFBP5* expression were also shown to be inversely correlated. Their findings confirmed the role of hsa-miR-204-5p as a tumor suppressor in PTC and revealed the potential use of this miRNA as a therapeutic agent in the treatment of PTC.

In an attempt to identify genes and pathways associated with mortality in PTC, Nilubol et al. (2011) performed genome-wide expression (GWE) analysis in 64 PTC patients and identified the oxidative phosphorylation pathway as one of the significantly perturbed pathways. In addition, Lee and colleagues, (2015a) also showed that the expression of OxPhos gene sets was significantly lower in primary PTC than in matched normal thyroid tissue. Our findings revealed a similar trend with OxPhos genes being significantly downregulated in PTCs versus normal thyroid tissues as well as in PTC LNM-P versus PTC LNN. However, significant enrichment of OxPhos pathway was only observed in PTC LNM-P compared to PTC LNN. Alteration in metabolic processes has been considered as an indispensable component of malignant transformation (Lee et al., 2015a) thus the involvement of oxidative phosphorylation in LNM in PTC necessitates further investigation.

Oxidative phosphorylation is a process whereby an adenosine triphosphate (ATP) is produced as a result of electrons transfer from nicotinamide adenine dinucleotide (NADH) or flavin-adenine dinucleotide (FADH_2_) to oxygen by a series of electron carriers (Berg, Tymoczko & Stryer, 2002). The thyroid gland is an endocrine organ with a high energy consumption and oxidative processes are crucial for thyroid hormone synthesis (Lee et al., 2015b). The mitochondria is responsible for providing 90% of the cellular energy necessary for various biological functions through oxidative phosphorylation and plays an important role in energy metabolism in the normal thyroid gland and in thyroid tumors (Kim et al., 2012). The mitochondria is involved in many cell signaling pathways by playing crucial roles in apoptosis, cell proliferation and cellular Ca^2+^ homeostasis (Rustin, 2002). Mitochondrial DNA (mtDNA) content was shown to be higher in PTC compared to the paired normal DNA and in normal controls (Mambo et al., 2005). Despite advancement in the elucidation of molecular events underlying thyroid carcinogenesis in the last decade, the function and nature of energy metabolism in thyroid cancer remain unclear (Lee et al., 2015b).

In addition to oxidative phosphorylation, we also identified significant enrichment of other cancer-related pathways such as cell adhesion molecules (CAMs), leukocyte transendothelial migration and cytokine–cytokine receptor interaction pathways which were unique to PTC LNM-P versus PTC LNN. Interestingly, these pathways were not significantly enriched when PTCs (LNM-P and LNN) were compared to normal thyroid tissues. Taken together, it could be hypothesized that metastasis to the lymph node in PTC occurred via changes in the aforementioned pathways. However, some pathways in our analysis, such as valine, leucine and isoleucine degradation, could not be associated with oncogenesis or metastasis and may need further investigation.

Our integrated analysis revealed hsa-miR-301b’s presence in the top 20 circuits in both PTC LNM-P versus PTC LNN and PTC LNM-P versus normal thyroid but was absent in PTC LNN versus normal thyroid despite significant downregulation with modest fold change (log_2_ fold change of −0.3). Hsa-miR-301 is located in the intronic region of *SKA2* (spindle and kinetochore associated complex subunit 2) and belongs to the hsa-miR-130 microRNA precursor family (Cao et al., 2010). In contrast to our findings, hsa-miR-301 upregulation has been reported in various cancers of non-thyroid origins and given that a miRNA can act either as an oncomiR or tumor suppressor depending on the cellular context and tissue type (Garzon, Calin & Croce, 2009), this observation is not unexpected. There is no evidence of hsa-miR-301b dysregulation in PTC so far, but it was reported to be upregulated in follicular thyroid adenoma compared to normal thyroid tissue (Rossing et al., 2012). It is also upregulated in CRC without LNM in comparison to paracancerous control (Wang et al., 2010b). The inhibition of hsa-miR-301 decreased breast cancer cell proliferation, clonogenicity, migration, invasion, tamoxifen resistance, tumor growth and microvessel density, further establishing this miRNA as an oncomiR (Shi et al., 2011). *FOXF2*, *BBC3*, *PTEN*, and *COL2A1* were confirmed as its direct targets through luciferase reporter assays (Shi et al., 2011).

Transcription factors (TFs) are a group of proteins involved in the initiation of transcription and are important for the regulation of genes. Majority of oncogenes and tumor suppressor genes encode the TFs (Ell & Kang, 2013). Dysregulation of oncogenic or tumor suppressive TFs could influence multiple steps of the metastasis cascade, leading to cancer progression (Ell & Kang, 2013). The involvement of TFs in PTC has been investigated since decades ago and several thyroid-specific TFs have been identified (Guazzi et al., 1990; Fabbro et al., 1994). Most recently, the glioma-associated oncogene homolog 1 (GLI1) has been identified as a TF marker for LNM in PTC and it increases tumor aggressiveness via the Hedgehog signaling pathway (Lee et al., 2015c). The hepatic leukemia factor (HLF) is the only TF which appeared in the top 20 circuits of PTCs with or without LNM versus normal thyroid from our integrated analysis. On the other hand, REL was identified in the top 20 circuits only in PTC LNM-P in comparison to PTC LNN and will be discussed further in the following section.

The HLF is a transcription factor that facilitates thyroid hormone activation from the thyroid hormone receptor/retinoid X receptor heterodimer to hypoxia-inducible factor (HIF-1*α*) (Otto & Fandrey, 2008). Triiodothyronine (T3) indirectly increases HIF-1*α* mRNA by increasing the expression of HLF, subsequently initiating the transcription of HIF-1*α* transcription factor (Burrows et al., 2011). HIF is another transcription factor which acts under hypoxia and thus is active in a number of diseases associated with low oxygen environment including cancer (Burrows et al., 2011). In fact, the HIF-1*α* protein was differentially expressed in primary thyroid cancers associated with advanced stage; its expression was supressed in normal thyroid tissue and was highest in the most aggressive dedifferentiated anaplastic thyroid carcinomas (ATCs) (Hanada, Feng & Hemmings, 2004), supporting its role for thyroid tumor aggressiveness, progression as well as metastasis. In addition, we also identified a significant involvement of REL/NFkB in lymph node metastasis of PTC which is in concordance with previously published data (Du et al., 2006).

In summary, we found evidence of five miRNAs differentially expressed in PTC LNM-P. Enrichment analysis revealed that alteration in oxidative phosphorylation pathway could be a key event involved in the lymph node metastasis of PTC suggesting that manipulation of the energy metabolism processes may provide an alternative therapeutic target for tackling metastasis or recurrence. In addition, via the integrated analysis we discovered that hsa-miR-301b might be involved in promoting LNM in PTC via activation of HLF, HIF and REL/NFkB. As far as we know, the roles of these TFs have been explored in PTC; however the exact roles of this miRNA with these TFs in LNM in PTC have not been studied. Hence, further investigation is necessary for future research in order to completely unravel the mechanism of LNM in PTC.

##  Supplemental Information

10.7717/peerj.2119/supp-1 Table S1 TCGA sample IDs of 477 papillary thyroid cancer patients with microRNA and gene expression data

10.7717/peerj.2119/supp-2 Table S2 181 significantly deregulated miRNAs with respective log_2_ fold change and *p* value in PTC LNM-P versus PTC LNN

10.7717/peerj.2119/supp-3 Table S3 78 significantly deregulated miRNAs with respective log_2_ fold change and *p* value in PTCs (LNM-P and LNN) versus normal thyroid

10.7717/peerj.2119/supp-4 File S1 R script for miRNAseq differential analysis

10.7717/peerj.2119/supp-5 Table S4 407 significantly deregulated genes with respective log_2_ fold change and *p* value in PTC LNM-P versus PTC LNN

10.7717/peerj.2119/supp-6 File S2 R script for mRNAseq differential analysis

10.7717/peerj.2119/supp-7 Data S1 Raw counts generated from miRNAseq in validation experiment

10.7717/peerj.2119/supp-8 Figure S1 Heat Map of the 395 Differentially Expressed miRNAs in PTC LNM-P versus normal thyroid. (Student’s T-test with BH corrected *p* value ≤0.05)

10.7717/peerj.2119/supp-9 Figure S2 Heat Map of the 400 Differentially Expressed miRNAs in PTC LNN versus normal thyroid. (Student’s T-test with BH corrected *p* value ≤0.05)

10.7717/peerj.2119/supp-10 Figure S3 Top 20 mixed regulatory circuits, involving miRNAs, genes and TFs There are two types of mixed regulatory circuits; a TF regulating both a given miRNA and its target gene and a miRNA that regulates both a given TF and its regulated gene. (A) PTC LNM-P compared to PTC LNN. (B) PTC LNM-P compared to normal thyroid. (C) PTC LNN compared to normal thyroid.

## Additional Information and Declarations

### Data Availability

The following information was supplied regarding data availability: The raw data generated from the validation study has been supplied as Data S1.

## References

[ref-1] Acibucu F, Dökmetaş HS, Tutar Y, Elagoz S, Kilicli F (2014). Correlations between the expression levels of microRNA146b, 221, 222 and p27Kip1 protein mRNA and the clinicopathologic parameters in papillary thyroid cancers. Experimental and Clinical Endocrinology &amp; Diabetes.

[ref-2] Edge SB, Byrd DR, Compton CC, Fritz AG, Greene FL, Trotti A, AJCC (2010). Thyroid. AJCC cancer staging manual.

[ref-3] Alexiou P, Maragkakis M, Papadopoulos GL, Reczko M, Hatzigeorgiou AG (2009). Lost in translation: an assessment and perspective for computational microRNA target identification. Bioinformatics.

[ref-4] Bao W, Wang HH, Tian FJ, He XY, Qiu MT, Wang JY, Zhang HJ, Wang LH1, Wan XP (2013). A TrkB-STAT3-miR-204-5p regulatory circuitry controls proliferation and invasion of endometrial carcinoma cells. Molecular Cancer.

[ref-5] Bartel DP (2009). Micrornas: target recognition and regulatory functions. Cell.

[ref-6] Beattie J, Allan GJ, Lochrie JD, Flint DJ (2006). Insulin-like growth factor-binding protein-5 (IGFBP-5): a critical member of the IGF axis. Biochemical Journal.

[ref-7] Berg JM, Tymoczko JL, Stryer L (2002). Biochemistry.

[ref-8] Bisognin A, Sales G, Coppe A, Bortoluzzi S, Romualdi C (2012). MAGIA^2^: from miRNA and genes expression data integrative analysis to microRNA-transcription factor mixed regulatory circuits (2012 update). Nucleic Acids Research.

[ref-9] Burrows N, Babur M, Resch J, Williams KJ, Brabant G (2011). Hypoxia-inducible factor in thyroid carcinoma. Journal of Thyroid Research.

[ref-10] Cady B, Rosai R (1988). An expanded view of risk group definition in differentiated thyroid carcinoma. Surgery.

[ref-11] Cancer Genome Atlas Research Network (2014). Integrated genomic characterization of papillary thyroid carcinoma. Cell.

[ref-12] Cao G, Huang B, Liu Z, Zhang J, Xu H, Xia W, Li J, Li S, Chen L, Ding H, Zhao Q, Fan M, Shen B, Shao N (2010). Intronic miR-301 feedback regulates its host gene, ska2, in A549 cells by targeting MEOX2 to affect ERK/CREB pathways. Biochemical and Biophysical Research Communications.

[ref-13] Chen YT, Kitabayashi N, Zhou XK, Fahey TJ, Scognamiglio T (2008). MicroRNA analysis as a potential diagnostic tool for papillary thyroid carcinoma. Modern Pathology.

[ref-14] Chou CK, Chen RF, Chou FF, Chang HW, Chen YJ, Lee YF, Yang KD, Cheng JT, Huang CC, Liu RT (2010). miR-146b is highly expressed in adult papillary thyroid carcinomas with high risk features including extrathyroidal invasion and the BRAF(V600E) mutation. Thyroid.

[ref-15] Chou CK, Yang KD, Chou FF, Huang CC, Lan YW, Lee YF, Kang HY, Liu RT (2013). Prognostic implications of miR-146b expression and its functional role in papillary thyroid carcinoma. Journal of Clinical Endocrinology and Metabolism.

[ref-16] Cordero F, Beccuti M, Arigoni M, Donatelli S, Calogero RA (2012). Optimizing a massive parallel sequencing workflow for quantitative miRNA expression analysis. PLoS ONE.

[ref-17] De Carvalho AC, Scapulatempo-Neto C, Maia DC, Evangelista AF, Morini MA, Carvalho AL, Vettore AL (2015). Accuracy of microRNAs as markers for the detection of neck lymph node metastases in patients with head and neck squamous cell carcinoma. BMC Medicine.

[ref-18] Deng X, Wu B, Xiao K, Kang J, Xie J, Zhang X, Fan Y (2015). MiR-146b-5p promotes metastasis and induces epithelial-mesenchymal transition in thyroid cancer by targeting ZNRF3. Cellular Physiology and Biochemistry.

[ref-19] Du ZX, Zhang HY, Gao DX, Wang HQ, Li YJ, Liu GL (2006). Significance of VEGF and NF-*κ*B expression in thyroid carcinoma. Chinese Journal of Clinical Oncology.

[ref-20] Ell B, Kang Y (2013). Transcriptional control of cancer metastasis. Trends in Cell Biology.

[ref-21] Fabbro D, Di Loreto C, Beltrami CA, Belfiore A, Di Lauro R, Damante G (1994). Expression of thyroid-specific transcription factors TTF-1 and PAX-8 in human thyroid neoplasms. Cancer Research.

[ref-22] Fassina A, Cappellesso R, Simonato F, Siri M, Ventura L, Tosato F, Busund LT, Pelizzo MR, Fassan M (2014). A 4-MicroRNA signature can discriminate primary lymphomas from anaplastic carcinomas in thyroid cytology smears. Cancer Cytopathology.

[ref-23] Friard O, Re A, Taverna D, De Bortoli M, Corá D (2010). CircuitsDB: a database of mixed microRNA/transcription factor feed-forward regulatory circuits in human and mouse. BMC Bioinformatics.

[ref-24] Garzon R, Calin GA, Croce CM (2009). MicroRNAs in cancer. Annual Review of Medicine.

[ref-25] Gentleman RC, Carey VJ, Bates DM, Bolstad B, Dettling M, Dudoit S, Ellis B, Gautier L, Ge Y, Gentry J, Hornik K, Hothorn T, Huber W, Iacus S, Irizarry R, Leisch F, Li C, Maechler M, Rossini AJ, Sawitzki G, Smith C, Smyth G, Tierney L, Yang JY, Zhang J (2004). Bioconductor: open software development for computational biology and bioinformatics. Genome Biology.

[ref-26] Geraldo MV, Yamashita AS, Kimura ET (2012). MicroRNA miR-146b-5p regulates signal transduction of TGF-*β* by repressing SMAD4 in thyroid cancer. Oncogene.

[ref-27] Gowrishankar B, Ibragimova I, Zhou Y, Slifker MJ, Devarajan K, Al-Saleem T, Uzzo RG, Cairns P (2014). MicroRNA expression signatures of stage, grade, and progression in clear cell RCC. Cancer Biology &amp; Therapy.

[ref-28] Guazzi S, Price M, De Felice M, Damante G, Mattei MG, Di Lauro R (1990). Thyroid nuclear factor 1 (TTF-1) contains a homeodomain and displays a novel DNA binding specificity. The EMBO Journal.

[ref-29] Hanada M, Feng J, Hemmings BA (2004). Structure, regulation and function of PKB/AKT–a major therapeutic target. Biochimica et Biophysica ACTA/General Subjects.

[ref-30] Hao HX, Xie Y, Zhang Y, Charlat O, Oster E, Avello M, Lei H, Mickanin C, Liu D, Ruffner H, Mao X, Ma Q, Zamponi R, Bouwmeester T, Finan PM, Kirschner MW, Porter JA, Serluca FC, Cong F (2012). ZNRF3 promotes Wnt receptor turnover in an R-spondin-sensitive manner. Nature.

[ref-31] Hay ID, Bergstrahl EJ, Goellner JR, Ebersold JR, Grant CS (1993). Predicting outcome in papillary thyroid carcinoma: development of a reliable prognostic scoring system in a cohort of 1779 patients surgically treated at one institution during 1940 through 1989. Surgery.

[ref-32] Hay ID, Grant CS, Taylor WF, MaConahey WM (1987). Ipsilateral lobectomy versus bilateral lobar resection in papillary thyroid carcinoma: a retrospective analysis of survical outcome using a novel prognostic scoring system. Surgery.

[ref-33] He H, Jazdzewski K, Li W, Liyanarachchi S, Nagy R, Volinia S, Calin GA, Liu CG, Franssila K, Suster S, Kloos RT, Croce CM, De la Chapelle A (2005). The role of microRNA genes in papillary thyroid carcinoma. Proceedings of the National Academy of Sciences of the United States of America.

[ref-34] Huang KH, Lan YT, Fang WL, Chen JH, Lo SS, Li AF, Chiou SH, Wu CW, Shyr YM (2015). The correlation between miRNA and lymph node metastasis in gastric cancer. BioMed Research International.

[ref-35] Huang DW, Sherman BT, Lempicki RA (2009a). Bioinformatics enrichment tools: paths toward the comprehensive functional analysis of large gene lists. Nucleic Acids Research.

[ref-36] Huang DW, Sherman BT, Lempicki RA (2009b). Systematic and integrative analysis of large gene lists using DAVID bioinformatics resources. Nature Protocols.

[ref-37] Iorio MV, Croce CM (2012). Microrna dysregulation in cancer: diagnostics, monitoring and therapeutics. A comprehensive review. EMBO Molecular Medicine.

[ref-38] Ito Y, Fukushima M, Tomoda C, Inoue H, Kihara M, Higashiyama T, Uruno T, Takamura Y, Miya A, Kobayashi K, Matsuzuka F, Miyauchi A (2009). Prognosis of patients with papillary thyroid carcinoma having clinically apparent metastasis to the lateral compartment. Endocrine Journal.

[ref-39] Kanehisa M, Goto S (2000). KEGG: Kyoto Encyclopedia of Genes and Genomes. Nucleic Acids Research.

[ref-40] Kanehisa M, Sato Y, Kawashima M, Furumichi M, Tanabe M (2016). KEGG as a reference resource for gene and protein annotation. Nucleic Acids Research.

[ref-41] Kim SJ, Kwon MC, Ryu MJ, Chung HK, Tadi S, Kim YK, Kim JM, Lee SH, Park JH, Kweon GR, Ryu SW, Jo YS, Lee CH, Hatakeyama H, Goto Y, Yim YH, Chung J, Kong YY, Shong M (2012). CRIF1 is essential for the synthesis and insertion of oxidative phosphorylation polypeptides in the mammalian mitochondrial membrane. Cell Metabolism.

[ref-42] Lee J, Chang JY, Kang YE, Yi S, Lee MH, Joung KH, Kim KS, Shong M (2015b). Mitochondrial energy metabolism and thyroid cancers. Endocrinology Metabolism.

[ref-43] Lee J, Jeong S, Lee CR, Ku CR, Kang SW, Jeong JJ, Nam KH, Shin DY, Chung WY, Lee EJ, Jo YS (2015c). GLI1 transcription factor affects tumor aggressiveness in patients with papillary thyroid cancers. Medicine.

[ref-44] Lee H, Kim KR, Cho NH, Hong SR, Jeong H, Kwon SY, Park KH, An HJ, Kim TH, Kim I, Yoon HK, Suh KS, Min KO, Choi HJ, Park JY, Yoo CW, Lee YS, Lee HJ, Lee WS, Park CS, Lee Y (2014). Gynecological pathology study group of the Korean society of pathologists. MicroRNA expression profiling and Notch1 and Notch2 expression in minimal deviation adenocarcinoma of uterine cervix. World Journal of Surgical Oncology.

[ref-45] Lee J, Seol MY, Jeong S, Lee CR, Ku CR, Kang SW, Jeong JJ, Shin DY, Nam KH, Lee EJ, Chung WY, Jo YS (2015a). A metabolic phenotype based on mitochondrial ribosomal protein expression as a predictor of lymph node metastasis in papillary thyroid carcinoma. Medicine.

[ref-46] Lee JC, Zhao JT, Clifton-Bligh RJ, Gill A, Gundara JS, Ip JC, Glover A, Sywak MS, Delbridge LW, Robinson BG, Sidhu SB (2013). MicroRNA-222 and microRNA-146b are tissue and circulating biomarkers of recurrent papillary thyroid cancer. Cancer.

[ref-47] Li B, Dewey CN (2011). RSEM: accurate transcript quantification from RNA-Seq data with or without a reference genome. BMC Bioinformatics.

[ref-48] Li W, Jin X, Zhang Q, Zhang G, Deng X, Ma L (2014). Decreased expression of miR-204 is associated with poor prognosis in patients with breast cancer. International Journal of Clinical and Experimental Pathology.

[ref-49] Liu L, Wang J, Li X, Ma J, Shi C, Zhu H, Xi Q, Zhang J, Zhao X, Gu M (2015). MiR-204-5p suppresses cell proliferation by inhibiting IGFBP5 in papillary thyroid carcinoma. Biochemical and Biophysical Research Communications.

[ref-50] Ma L, Huang Y, Zhu W, Zhou S, Zhou J, Zeng F, Liu X, Zhang Y, Yu J (2011). An integrated analysis of miRNA and mRNA expressions in non-small cell lung cancers. PLoS ONE.

[ref-51] Mambo E, Chatterjee A, Xing M, Tallini G, Haugen BR, Yeung SC, Sukumar S, Sidransky D (2005). Tumor-specific changes in mtDNA content in human cancer. International Journal of Cancer.

[ref-52] Maragkakis M, Reczko M, Simossis VA, Alexiou P, Papadopoulos GL, Dalamagas T, Giannopoulos G, Goumas G, Koukis E, Kourtis K, Vergoulis T, Koziris N, Sellis T, Tsanakas P, Hatzigeorgiou AG (2009). DIANA-microT web server: elucidating microRNA functions through target prediction. Nucleic Acids Research.

[ref-53] Nilubol N, Sukchotrat C, Zhang L, He M, Kebebew E (2011). Molecular pathways associated with mortality in papillary thyroid cancer. Surgery.

[ref-54] Nunez-Iglesias J, Liu CC, Morgan TE, Finch CE, Zhou XJ (2010). Joint genome-wide profiling of miRNA and mRNA expression in Alzheimer’s disease cortex reveals altered miRNA regulation. PLoS ONE.

[ref-55] Otto T, Fandrey J (2008). Thyroid hormone induces hypoxia-inducible factor 1alpha gene expression through thyroid hormone receptor beta/retinoid x receptor alpha-dependent activation of hepatic leukemia factor. Endocrinology.

[ref-56] Pallante P, Visone R, Ferracin M, Ferraro A, Berlingieri MT, Troncone G, Chiappetta G, Liu CG, Santoro M, Negrini M, Croce CM, Fusco A (2006). MicroRNA deregulation in human thyroid papillary carcinomas. Endocrine-Related Cancer.

[ref-57] Pizzini S, Bisognin A, Mandruzzato S, Biasiolo M, Facciolli A, Perilli L, Rossi E, Esposito G, Rugge M, Pilati P, Mocellin S, Nitti D, Bortoluzzi S, Zanovello P (2013). Impact of microRNAs on regulatory networks and pathways in human colorectal carcinogenesis and development of metastasis. BMC Genomics.

[ref-58] R Development Core Team (2008). R: a language and environment for statistical computing.

[ref-59] Rossing M, Borup R, Henao R, Winther O, Vikesaa J, Niazi O, Godballe C, Krogdahl A, Glud M, Hjort-Sørensen C, Kiss K, Bennedbæk FN, Nielsen FC (2012). Down-regulation of microRNAs controlling tumourigenic factors in follicular thyroid carcinoma. Journal of Molecular Endocrinology.

[ref-60] Rustin P (2002). Mitochondria, from cell death to proliferation. Nature Genetics.

[ref-61] Schvartz C, Bonnetain F, Dabakuyo S, Gauthier M, Cueff A, Fieffé S, Pochart JM, Cochet I, Crevisy E, Dalac A, Papathanassiou D, Toubeau M (2012). Impact on overall survival of radioactive iodine in low-risk differentiated thyroid cancer patients. Journal of Clinical Endocrinology and Metabolism.

[ref-62] Shi W, Gerster K, Alajez NM, Tsang J, Waldron L, Pintilie M, Hui AB, Sykes J, P'ng C, Miller N, McCready D, Fyles A, Liu FF (2011). MicroRNA-301 mediates proliferation and invasion in human breast cancer. Cancer Research.

[ref-63] Slaby O, Svoboda M, Fabian P, Smerdova T, Knoflickova D, Bednarikova M, Nenutil R, Vyzula R (2007). Altered expression of miR-21, miR-31, miR-143 and miR-145 is related to clinicopathologic features of colorectal cancer. Oncology.

[ref-64] Sobin LH, Wittekind Ch (2002). UICC: TNM classification of malignant tumors.

[ref-65] Sun Y, Yu S, Liu Y, Wang F, Liu Y, Xiao H (2013). Expression of miRNAs in papillary thyroid carcinomas is associated with BRAF mutation and clinicopathological features in Chinese patients. International Journal of Endocrinology.

[ref-66] Swierniak M, Wojcicka A, Czetwertynska M, Stachlewska E, Maciag M, Wiechno W, Gornicka B, Bogdanska M, Koperski L, De la Chapelle A, Jazdzewski K (2013). In-depth characterization of the microRNA transcriptome in normal thyroid and papillary thyroid carcinoma. Journal of Clinical Endocrinology and Metabolism.

[ref-67] Tetzlaff MT, Liu A, Xu X, Master SR, Baldwin DA, Tobias JW, Livolsi VA, Baloch ZW (2007). Differential expression of miRNAs in papillary thyroid carcinoma compared to multinodular goiter using formalin fixed paraffin embedded tissues. Endocrine Pathology.

[ref-68] Wang J, Lu M, Qiu C, Cui Q (2010a). TransmiR: a transcription factor--microRNA regulation database. Nucleic Acids Research.

[ref-69] Wang YX, Zhang XY, Zhang BF, Yang CQ, Chen XM, Gao HJ (2010b). Initial study of microRNA expression profiles of colonic cancer without lymph node metastasis. Journal of Digestive Diseases.

[ref-70] Wojtas B, Ferraz C, Stokowy T, Hauptmann S, Lange D, Dralle H, Musholt T, Jarzab B, Paschke R, Eszlinger M (2014). Differential miRNA expression defines migration and reduced apoptosis in follicular thyroid carcinomas. Molecular and Cellular Endocrinology.

[ref-71] Yan LX, Huang XF, Shao Q, Huang MY, Deng L, Wu QL, Zeng YX, Shao JY (2008). MicroRNA miR-21 overexpression in human breast cancer is associated with advanced clinical stage, lymph node metastasis and patient poor prognosis. RNA.

[ref-72] Yang Z, Yuan Z, Fan Y, Deng X, Zheng Q (2013). Integrated analyses of microRNA and mRNA expression profiles in aggressive papillary thyroid carcinoma. Molecular Medicine Reports.

[ref-73] Yin Y, Zhang B, Wang W, Fei B, Quan C, Zhang J, Song M, Bian Z, Wang Q, Ni S, Hu Y, Mao Y, Zhou L, Wang Y, Yu J, Du X, Hua D, Huang Z (2014). miR-204-5p inhibits proliferation and invasion and enhances chemotherapeutic sensitivity of colorectal cancer cells by downregulating RAB22a. Clinical Cancer Research.

[ref-74] Yip L, Kelly L, Shuai Y, Armstrong MJ, Nikiforov YE, Carty SE, Nikiforova MN (2011). MicroRNA signature distinguishes the degree of aggressiveness of papillary thyroid carcinoma. Annals of Surgical Oncology.

