# Supplementary material for: Integrated microRNA, gene expression and transcription factors signature in papillary thyroid cancer with lymph node metastasis
Source: PeerJ. 2016 Jun 15;4:e2119. doi: 10.7717/peerj.2119 (PMC4918724; doi:10.7717/peerj.2119)
Supplement: Table S1 [file peerj-04-2119-s001.docx]

Supplementary Table 1: TCGA sample IDs of 477 papillary thyroid cancer patients with microRNA and gene expression data

| **Sample IDs** | **Sample IDs** | **Sample IDs** | **Sample IDs** | **Sample IDs** |
| --- | --- | --- | --- | --- |
| \| TCGA-BJ-A0ZB \| \| --- \| \| TCGA-BJ-A0ZH \| \| TCGA-DE-A0Y3 \| \| TCGA-DE-A3KN \| \| TCGA-DE-A4MB \| \| TCGA-DE-A4MC \| \| TCGA-DE-A4MD \| \| TCGA-DJ-A1QD \| \| TCGA-DJ-A2Q1 \| \| TCGA-DJ-A2Q5 \| \| TCGA-DJ-A2Q6 \| \| TCGA-DJ-A2QB \| \| TCGA-DJ-A3UO \| \| TCGA-DJ-A3V0 \| \| TCGA-DJ-A3V8 \| \| TCGA-DJ-A3V9 \| \| TCGA-DJ-A3VF \| \| TCGA-DJ-A4UQ \| \| TCGA-DJ-A4UR \| \| TCGA-DJ-A4UW \| \| TCGA-DJ-A4V5 \| \| TCGA-DO-A1K0 \| \| TCGA-DO-A2HM \| \| TCGA-E3-A3E5 \| \| TCGA-E8-A415 \| \| TCGA-E8-A436 \| \| TCGA-EL-A3CM \| \| TCGA-EL-A3CO \| \| TCGA-EL-A3CR \| \| TCGA-EL-A3CV \| \| TCGA-EL-A3CY \| \| TCGA-EM-A1CT \| \| TCGA-EM-A2CK \| \| TCGA-EM-A2CS \| \| TCGA-EM-A2OX \| \| TCGA-EM-A2P0 \| \| TCGA-EM-A2P1 \| \| TCGA-EM-A3AN \| \| TCGA-EM-A3AO \| \| TCGA-EM-A3FJ \| \| TCGA-EM-A3FK \| \| TCGA-EM-A3FM \| \| TCGA-EM-A3FQ \| \| TCGA-EM-A3SU \| \| TCGA-EM-A4FM \| \| TCGA-EM-A4FN \| \| TCGA-EM-A22K \| \| TCGA-EM-A22P \| \| TCGA-ET-A3BT \| \| TCGA-ET-A25L \| \| TCGA-ET-A40Q \| \| TCGA-FE-A231 \| \| TCGA-FK-A3S3 \| \| TCGA-FK-A3SG \| \| TCGA-FY-A3R6 \| \| TCGA-FY-A40L \| \| TCGA-GE-A2C6 \| \| TCGA-H2-A2K9 \| \| TCGA-IM-A3U2 \| \| TCGA-IM-A4EB \| \| TCGA-J8-A3O1 \| \| TCGA-J8-A3O2 \| \| TCGA-J8-A3YH \| \| TCGA-J8-A4HY \| \| TCGA-KS-A4I5 \| \| TCGA-MK-A4N6 \| \| TCGA-BJ-A0Z5 \| \| TCGA-BJ-A0ZJ \| \| TCGA-BJ-A3PU \| \| TCGA-BJ-A4O8 \| \| TCGA-BJ-A28T \| \| TCGA-BJ-A28X \| \| TCGA-BJ-A28Z \| \| TCGA-BJ-A45J \| \| TCGA-BJ-A290 \| \| TCGA-DE-A0XZ \| \| TCGA-DE-A0Y2 \| \| TCGA-DE-A4MA \| \| TCGA-DJ-A1QH \| \| TCGA-DJ-A2PQ \| \| TCGA-DJ-A2PR \| \| TCGA-DJ-A2PS \| \| TCGA-DJ-A2PW \| \| TCGA-DJ-A2PY \| \| TCGA-DJ-A2Q3 \| \| TCGA-DJ-A2Q4 \| \| TCGA-DJ-A2Q7 \| \| TCGA-DJ-A2Q9 \| \| TCGA-DJ-A3UQ \| \| TCGA-DJ-A3UR \| \| TCGA-DJ-A3V2 \| \| TCGA-DJ-A3V4 \| \| TCGA-DJ-A3VA \| \| TCGA-DJ-A3VB \| \| TCGA-DJ-A3VD \| | \| TCGA-DJ-A3VJ \| \| --- \| \| TCGA-E3-A3DY \| \| TCGA-E3-A3E2 \| \| TCGA-E8-A2JQ \| \| TCGA-E8-A417 \| \| TCGA-EL-A3CU \| \| TCGA-EL-A3CZ \| \| TCGA-EL-A3GP \| \| TCGA-EL-A3TB \| \| TCGA-EM-A1CU \| \| TCGA-EM-A2CQ \| \| TCGA-EM-A2CU \| \| TCGA-EM-A3AJ \| \| TCGA-EM-A3AR \| \| TCGA-EM-A3FR \| \| TCGA-EM-A3O3 \| \| TCGA-EM-A3SX \| \| TCGA-EM-A22O \| \| TCGA-ET-A2MX \| \| TCGA-ET-A2N0 \| \| TCGA-ET-A3BO \| \| TCGA-ET-A3BP \| \| TCGA-ET-A3BS \| \| TCGA-ET-A3BU \| \| TCGA-ET-A3BV \| \| TCGA-ET-A3BX \| \| TCGA-ET-A3DR \| \| TCGA-ET-A3DT \| \| TCGA-ET-A3DU \| \| TCGA-ET-A25J \| \| TCGA-ET-A25K \| \| TCGA-ET-A25N \| \| TCGA-ET-A25O \| \| TCGA-ET-A39K \| \| TCGA-ET-A40R \| \| TCGA-ET-A40T \| \| TCGA-FE-A3PB \| \| TCGA-FE-A230 \| \| TCGA-FK-A3SB \| \| TCGA-FK-A3SE \| \| TCGA-FK-A3SH \| \| TCGA-FK-A4UB \| \| TCGA-FY-A3NN \| \| TCGA-FY-A3R7 \| \| TCGA-FY-A3YR \| \| TCGA-FY-A4B3 \| \| TCGA-H2-A421 \| \| TCGA-IM-A3EB \| \| TCGA-IM-A41Y \| \| TCGA-IM-A420 \| \| TCGA-J8-A3NZ \| \| TCGA-J8-A3YD \| \| TCGA-J8-A3YG \| \| TCGA-J8-A42S \| \| TCGA-KS-A4IC \| \| TCGA-L6-A4EQ \| \| TCGA-L6-A4EU \| \| TCGA-BJ-A0YZ \| \| TCGA-BJ-A0Z0 \| \| TCGA-BJ-A0Z2 \| \| TCGA-BJ-A0Z3 \| \| TCGA-BJ-A0Z9 \| \| TCGA-BJ-A0ZA \| \| TCGA-BJ-A0ZC \| \| TCGA-BJ-A0ZE \| \| TCGA-BJ-A0ZG \| \| TCGA-BJ-A2NA \| \| TCGA-BJ-A3PR \| \| TCGA-BJ-A3PT \| \| TCGA-BJ-A4O9 \| \| TCGA-BJ-A18Y \| \| TCGA-BJ-A28R \| \| TCGA-BJ-A28V \| \| TCGA-BJ-A28W \| \| TCGA-BJ-A45C \| \| TCGA-BJ-A45D \| \| TCGA-BJ-A45E \| \| TCGA-BJ-A45F \| \| TCGA-BJ-A45H \| \| TCGA-BJ-A45I \| \| TCGA-BJ-A45K \| \| TCGA-BJ-A190 \| \| TCGA-BJ-A191 \| \| TCGA-BJ-A291 \| \| TCGA-CE-A3ME \| \| TCGA-CE-A481 \| \| TCGA-DJ-A1QE \| \| TCGA-DJ-A1QF \| \| TCGA-DJ-A1QG \| \| TCGA-DJ-A1QI \| \| TCGA-DJ-A1QL \| \| TCGA-DJ-A1QM \| \| TCGA-DJ-A1QN \| \| TCGA-DJ-A1QO \| \| TCGA-DJ-A1QQ \| | \| TCGA-DJ-A2PN \| \| --- \| \| TCGA-DJ-A2PO \| \| TCGA-DJ-A2PP \| \| TCGA-DJ-A2PT \| \| TCGA-DJ-A2PU \| \| TCGA-DJ-A2PV \| \| TCGA-DJ-A2PX \| \| TCGA-DJ-A2PZ \| \| TCGA-DJ-A2Q0 \| \| TCGA-DJ-A2Q2 \| \| TCGA-DJ-A2QA \| \| TCGA-DJ-A2QC \| \| TCGA-DJ-A3UK \| \| TCGA-DJ-A3UM \| \| TCGA-DJ-A3UN \| \| TCGA-DJ-A3UP \| \| TCGA-DJ-A3US \| \| TCGA-DJ-A3UT \| \| TCGA-DJ-A3UU \| \| TCGA-DJ-A3UW \| \| TCGA-DJ-A3UX \| \| TCGA-DJ-A3UY \| \| TCGA-DJ-A3UZ \| \| TCGA-DJ-A3V3 \| \| TCGA-DJ-A3V5 \| \| TCGA-DJ-A3V7 \| \| TCGA-DJ-A3VE \| \| TCGA-DJ-A3VG \| \| TCGA-DJ-A3VL \| \| TCGA-DJ-A3VM \| \| TCGA-DJ-A4UL \| \| TCGA-DJ-A4UT \| \| TCGA-DJ-A4V0 \| \| TCGA-DJ-A4V2 \| \| TCGA-DJ-A4V4 \| \| TCGA-DJ-A13M \| \| TCGA-DJ-A13O \| \| TCGA-DJ-A13P \| \| TCGA-DJ-A13R \| \| TCGA-DJ-A13S \| \| TCGA-DJ-A13T \| \| TCGA-DJ-A13U \| \| TCGA-DJ-A13W \| \| TCGA-DJ-A13X \| \| TCGA-E3-A3DZ \| \| TCGA-E3-A3E0 \| \| TCGA-E3-A3E1 \| \| TCGA-E3-A3E3 \| \| TCGA-E8-A2EA \| \| TCGA-E8-A3X7 \| \| TCGA-E8-A44K \| \| TCGA-E8-A44M \| \| TCGA-E8-A242 \| \| TCGA-E8-A413 \| \| TCGA-E8-A414 \| \| TCGA-E8-A416 \| \| TCGA-E8-A418 \| \| TCGA-E8-A432 \| \| TCGA-E8-A433 \| \| TCGA-E8-A434 \| \| TCGA-E8-A438 \| \| TCGA-EL-A3CN \| \| TCGA-EL-A3CS \| \| TCGA-EL-A3CX \| \| TCGA-EL-A3D1 \| \| TCGA-EL-A3GO \| \| TCGA-EL-A3GQ \| \| TCGA-EL-A3GV \| \| TCGA-EL-A3GW \| \| TCGA-EL-A3H1 \| \| TCGA-EL-A3H2 \| \| TCGA-EL-A3MW \| \| TCGA-EL-A3T1 \| \| TCGA-EL-A3T3 \| \| TCGA-EL-A3T6 \| \| TCGA-EL-A3T7 \| \| TCGA-EL-A3T8 \| \| TCGA-EL-A3TA \| \| TCGA-EL-A3ZG \| \| TCGA-EL-A3ZQ \| \| TCGA-EL-A3ZR \| \| TCGA-EL-A3ZT \| \| TCGA-EL-A4JV \| \| TCGA-EL-A4JX \| \| TCGA-EL-A4K2 \| \| TCGA-EL-A4K9 \| \| TCGA-EL-A4KG \| \| TCGA-EL-A4KH \| \| TCGA-EL-A4KI \| \| TCGA-EM-A1CS \| \| TCGA-EM-A1CV \| \| TCGA-EM-A1CW \| \| TCGA-EM-A1YC \| \| TCGA-EM-A1YD \| \| TCGA-EM-A1YE \| | \| TCGA-EM-A2CN \| \| --- \| \| TCGA-EM-A2CP \| \| TCGA-EM-A2CR \| \| TCGA-EM-A2CT \| \| TCGA-EM-A2OV \| \| TCGA-EM-A2OY \| \| TCGA-EM-A2OZ \| \| TCGA-EM-A2P2 \| \| TCGA-EM-A3AK \| \| TCGA-EM-A3AL \| \| TCGA-EM-A3AQ \| \| TCGA-EM-A3FN \| \| TCGA-EM-A3FO \| \| TCGA-EM-A3FP \| \| TCGA-EM-A3O8 \| \| TCGA-EM-A3O9 \| \| TCGA-EM-A3OA \| \| TCGA-EM-A3OB \| \| TCGA-EM-A3ST \| \| TCGA-EM-A3SZ \| \| TCGA-EM-A4FO \| \| TCGA-EM-A4FV \| \| TCGA-EM-A4G1 \| \| TCGA-EM-A22I \| \| TCGA-EM-A22J \| \| TCGA-EM-A22L \| \| TCGA-EM-A22M \| \| TCGA-EM-A22N \| \| TCGA-EM-A22Q \| \| TCGA-ET-A2MZ \| \| TCGA-ET-A2N4 \| \| TCGA-ET-A3BQ \| \| TCGA-ET-A3DQ \| \| TCGA-ET-A3DS \| \| TCGA-ET-A3DV \| \| TCGA-ET-A4KN \| \| TCGA-ET-A25G \| \| TCGA-ET-A25I \| \| TCGA-ET-A25P \| \| TCGA-ET-A39L \| \| TCGA-ET-A39O \| \| TCGA-ET-A39P \| \| TCGA-ET-A39R \| \| TCGA-ET-A39S \| \| TCGA-ET-A39T \| \| TCGA-ET-A40P \| \| TCGA-ET-A40S \| \| TCGA-FE-A3PA \| \| TCGA-FE-A3PD \| \| TCGA-FE-A23A \| \| TCGA-FE-A238 \| \| TCGA-FE-A239 \| \| TCGA-FK-A3SD \| \| TCGA-FY-A2QD \| \| TCGA-FY-A3I4 \| \| TCGA-FY-A3I5 \| \| TCGA-FY-A3NM \| \| TCGA-FY-A3NP \| \| TCGA-FY-A3R9 \| \| TCGA-FY-A3TY \| \| TCGA-FY-A3W9 \| \| TCGA-FY-A40K \| \| TCGA-FY-A40M \| \| TCGA-FY-A40N \| \| TCGA-H2-A3RH \| \| TCGA-H2-A3RI \| \| TCGA-H2-A26U \| \| TCGA-H2-A422 \| \| TCGA-IM-A3ED \| \| TCGA-IM-A3U3 \| \| TCGA-IM-A41Z \| \| TCGA-J8-A3O0 \| \| TCGA-KS-A4I1 \| \| TCGA-KS-A4I7 \| \| TCGA-KS-A4I9 \| \| TCGA-KS-A4ID \| \| TCGA-KS-A41I \| \| TCGA-KS-A41L \| \| TCGA-L6-A4EP \| \| TCGA-MK-A4N7 \| \| TCGA-BJ-A45G \| \| TCGA-CE-A3MD \| \| TCGA-CE-A13K \| \| TCGA-CE-A27D \| \| TCGA-CE-A482 \| \| TCGA-CE-A483 \| \| TCGA-CE-A484 \| \| TCGA-CE-A485 \| \| TCGA-DE-A4M8 \| \| TCGA-E8-A419 \| \| TCGA-EL-A3GS \| \| TCGA-EL-A3GU \| \| TCGA-EL-A3GX \| \| TCGA-EL-A3GY \| \| TCGA-EL-A3H4 \| | \| TCGA-EL-A3H5 \| \| --- \| \| TCGA-EL-A3H7 \| \| TCGA-EL-A3H8 \| \| TCGA-EL-A3MZ \| \| TCGA-EL-A3N2 \| \| TCGA-EL-A3T0 \| \| TCGA-EL-A3T2 \| \| TCGA-EL-A3T9 \| \| TCGA-EL-A3ZH \| \| TCGA-EL-A3ZK \| \| TCGA-EL-A3ZL \| \| TCGA-EL-A3ZP \| \| TCGA-EL-A3ZS \| \| TCGA-EL-A4K1 \| \| TCGA-EL-A4K4 \| \| TCGA-EL-A4K6 \| \| TCGA-EL-A4K7 \| \| TCGA-EL-A4KD \| \| TCGA-EM-A4FF \| \| TCGA-EM-A4FQ \| \| TCGA-EM-A4FR \| \| TCGA-ET-A3DW \| \| TCGA-ET-A25M \| \| TCGA-FE-A3PC \| \| TCGA-FE-A22Z \| \| TCGA-FE-A234 \| \| TCGA-FE-A235 \| \| TCGA-FE-A236 \| \| TCGA-FE-A237 \| \| TCGA-FY-A3ON \| \| TCGA-J8-A3YE \| \| TCGA-J8-A3YF \| \| TCGA-J8-A4HW \| \| TCGA-KS-A4I3 \| \| TCGA-KS-A4IB \| \| TCGA-KS-A41F \| \| TCGA-KS-A41J \| \| TCGA-MK-A4N9 \| \| TCGA-BJ-A2N7 \| \| TCGA-BJ-A2N8 \| \| TCGA-BJ-A2N9 \| \| TCGA-BJ-A2NA \| \| TCGA-BJ-A3PR \| \| TCGA-BJ-A3PU \| \| TCGA-BJ-A28R \| \| TCGA-BJ-A28W \| \| TCGA-BJ-A28X \| \| TCGA-BJ-A290 \| \| TCGA-DO-A1JZ \| \| TCGA-E8-A2JQ \| \| TCGA-EL-A3GZ \| \| TCGA-EL-A3H1 \| \| TCGA-EL-A3H2 \| \| TCGA-EL-A3H7 \| \| TCGA-EL-A3MW \| \| TCGA-EL-A3MX \| \| TCGA-EL-A3MY \| \| TCGA-EL-A3N2 \| \| TCGA-EL-A3N3 \| \| TCGA-EL-A3T0 \| \| TCGA-EL-A3T1 \| \| TCGA-EL-A3T2 \| \| TCGA-EL-A3T3 \| \| TCGA-EL-A3T6 \| \| TCGA-EL-A3T7 \| \| TCGA-EL-A3T8 \| \| TCGA-EL-A3TA \| \| TCGA-EL-A3TB \| \| TCGA-EL-A3ZG \| \| TCGA-EL-A3ZH \| \| TCGA-EL-A3ZK \| \| TCGA-EL-A3ZL \| \| TCGA-EL-A3ZM \| \| TCGA-EL-A3ZO \| \| TCGA-EL-A3ZP \| \| TCGA-EL-A3ZQ \| \| TCGA-EL-A3ZR \| \| TCGA-EL-A3ZS \| \| TCGA-EL-A3ZT \| \| TCGA-EM-A1CS \| \| TCGA-EM-A1CT \| \| TCGA-EM-A1CU \| \| TCGA-EM-A1CV \| \| TCGA-EM-A1CW \| \| TCGA-EM-A1YC \| \| TCGA-EM-A3ST \| \| TCGA-ET-A2MX \| \| TCGA-ET-A2N5 \| \| TCGA-ET-A3DP \| \| TCGA-ET-A3DW \| \| TCGA-FY-A3TY \| \| TCGA-GE-A2C6 \| \| TCGA-H2-A2K9 \| \| TCGA-H2-A3RI \| \| TCGA-KS-A41I \| \| TCGA-KS-A41J \| \| TCGA-KS-A41L \| |

Samples excluded from analyses were TCGA-DJ-A2Q8 and TCGA-ET-A2N1 due to no RNAseq data available while TCGA-DJ-A13V has no miRNAseq data. There were ten patients with matched normal adjacent tissue expression data: TCGA-BJ-A28X, TCGA-BJ-A290, TCGA-BJ-A3PU, TCGA-E8-A2JQ, TCGA-EL-A3TB, TCGA-EM-A1CT, TCGA-EM-A1CU, TCGA-ET-A2MX, TCGA-GE-A2C6 and TCGA-H2-A2K9.
