# Supplementary material for: Integrated microRNA, gene expression and transcription factors signature in papillary thyroid cancer with lymph node metastasis
Source: PeerJ. 2016 Jun 15;4:e2119. doi: 10.7717/peerj.2119 (PMC4918724; doi:10.7717/peerj.2119)
Supplement: Table S2 [file peerj-04-2119-s002.docx]

Supplementary Table 2. 181 significantly deregulated miRNAs with respective log_2_ fold change and p value in PTC LNM-P versus PTC LNN.

| **miRNA ID** | **Mean expression in PTC LNM-P (Log_2_ normalized)** | **Mean expression in PTC LNN (Log_2_ normalized)** | **Log_2_ fold change** | **BH adj. p value** |
| --- | --- | --- | --- | --- |
| hsa-miR-146b | 14.64587325 | 12.94028888 | 1.7 | 1.41E-03 |
| hsa-miR-375 | 12.55935383 | 11.21346453 | 1.3 | 3.00E-02 |
| hsa-miR-31 | 6.410072121 | 5.389400224 | 1.0 | 4.59E-09 |
| hsa-miR-205 | 5.535422 | 4.573066125 | 1.0 | 1.39E-04 |
| hsa-miR-551b | 4.939622224 | 3.989969647 | 0.9 | 1.47E-02 |
| hsa-miR-222 | 9.021798706 | 8.15911148 | 0.9 | 2.19E-03 |
| hsa-miR-221 | 10.87797755 | 10.05621894 | 0.8 | 5.53E-03 |
| hsa-miR-127 | 7.264973488 | 6.525730237 | 0.7 | 5.01E-03 |
| hsa-miR-21 | 17.6578352 | 16.96668448 | 0.7 | 4.66E-03 |
| hsa-miR-134 | 5.416349285 | 4.77367003 | 0.6 | 9.55E-04 |
| hsa-miR-136 | 3.368669318 | 2.733977291 | 0.6 | 2.53E-03 |
| hsa-miR-511-2 | 2.944621616 | 2.318424001 | 0.6 | 4.67E-04 |
| hsa-miR-934 | 1.311895902 | 0.715928717 | 0.6 | 2.28E-03 |
| hsa-miR-379 | 6.786336089 | 6.193295301 | 0.6 | 1.23E-03 |
| hsa-miR-382 | 2.348791436 | 1.781023529 | 0.6 | 3.81E-02 |
| hsa-miR-223 | 6.800551408 | 6.241091802 | 0.6 | 3.34E-03 |
| hsa-miR-142 | 10.27492722 | 9.718690359 | 0.6 | 5.05E-04 |
| hsa-miR-511-1 | 2.888459574 | 2.335852983 | 0.6 | 3.29E-02 |
| hsa-miR-514-3 | 2.933059752 | 2.382112058 | 0.6 | 1.17E-04 |
| hsa-miR-514-2 | 2.924035828 | 2.384135387 | 0.5 | 1.74E-02 |
| hsa-miR-337 | 2.952487114 | 2.417066044 | 0.5 | 1.26E-03 |
| hsa-miR-381 | 3.371545856 | 2.849950685 | 0.5 | 2.95E-04 |
| hsa-miR-508 | 5.383138233 | 4.86743697 | 0.5 | 2.78E-04 |
| hsa-miR-514-1 | 2.938609921 | 2.425637659 | 0.5 | 3.72E-02 |
| hsa-miR-409 | 2.807144037 | 2.297262008 | 0.5 | 2.49E-05 |
| hsa-miR-199a-1 | 9.097440594 | 8.590942539 | 0.5 | 8.25E-04 |
| hsa-miR-675 | 2.368375508 | 1.864021282 | 0.5 | 1.35E-02 |
| hsa-miR-493 | 2.050434507 | 1.547741867 | 0.5 | 3.51E-02 |
| hsa-miR-199a-2 | 9.797116741 | 9.304467232 | 0.5 | 4.46E-03 |
| hsa-miR-199b | 10.04380645 | 9.553148225 | 0.5 | 1.12E-10 |
| hsa-miR-509-2 | 2.083791196 | 1.617431805 | 0.5 | 5.83E-03 |
| hsa-miR-758 | 2.337259798 | 1.872346194 | 0.5 | 1.77E-03 |
| hsa-miR-654 | 2.854859943 | 2.393053516 | 0.5 | 4.02E-06 |
| hsa-miR-509-3 | 2.326090969 | 1.871970388 | 0.5 | 3.36E-07 |
| hsa-miR-214 | 3.731525786 | 3.300579815 | 0.4 | 8.04E-03 |
| hsa-miR-889 | 2.060021083 | 1.642313204 | 0.4 | 7.44E-04 |
| hsa-miR-708 | 7.227367749 | 6.822927661 | 0.4 | 1.14E-03 |
| hsa-miR-509-1 | 2.057525058 | 1.655999799 | 0.4 | 4.73E-02 |
| hsa-miR-146a | 6.668855553 | 6.28325051 | 0.4 | 3.45E-03 |
| hsa-miR-539 | 1.328231172 | 0.949362545 | 0.4 | 2.71E-04 |
| hsa-miR-196a-1 | 1.338472805 | 0.962725633 | 0.4 | 7.62E-04 |
| hsa-miR-431 | 1.494609006 | 1.133577586 | 0.4 | 1.17E-04 |
| hsa-miR-154 | 1.351813714 | 0.994723318 | 0.4 | 3.70E-02 |
| hsa-miR-92b | 6.967672708 | 6.611517781 | 0.4 | 2.63E-03 |
| hsa-miR-369 | 1.639108375 | 1.288044176 | 0.4 | 1.26E-02 |
| hsa-miR-135b | 7.933581362 | 7.598230234 | 0.3 | 1.76E-03 |
| hsa-miR-411 | 1.544338843 | 1.211502902 | 0.3 | 2.26E-03 |
| hsa-miR-33b | 1.995425751 | 1.666100973 | 0.3 | 2.95E-03 |
| hsa-miR-3622a | 1.083479036 | 0.755248579 | 0.3 | 4.77E-06 |
| hsa-miR-410 | 1.930681094 | 1.603431556 | 0.3 | 4.75E-02 |
| hsa-miR-203 | 11.77307024 | 11.45813074 | 0.3 | 2.60E-02 |
| hsa-miR-944 | 0.976166273 | 0.66511832 | 0.3 | 3.09E-09 |
| hsa-miR-370 | 1.496018414 | 1.185020057 | 0.3 | 1.26E-03 |
| hsa-miR-19b-1 | 3.081394546 | 2.777190032 | 0.3 | 1.15E-02 |
| hsa-miR-27a | 10.64506259 | 10.34404914 | 0.3 | 8.25E-03 |
| hsa-miR-487b | 1.189315385 | 0.892352555 | 0.3 | 1.36E-10 |
| hsa-miR-224 | 4.416119978 | 4.119597139 | 0.3 | 4.57E-02 |
| hsa-miR-218-1 | 1.602487231 | 1.310825724 | 0.3 | 1.17E-02 |
| hsa-miR-495 | 1.407882896 | 1.123664575 | 0.3 | 2.82E-03 |
| hsa-miR-299 | 0.903359302 | 0.627142341 | 0.3 | 1.14E-03 |
| hsa-miR-485 | 1.073501616 | 0.7996811 | 0.3 | 6.87E-03 |
| hsa-miR-376c | 1.248733572 | 0.975084533 | 0.3 | 4.35E-04 |
| hsa-miR-29b-1 | 9.773111223 | 9.516071644 | 0.3 | 1.15E-07 |
| hsa-let-7i | 11.33528819 | 11.09021948 | 0.2 | 3.19E-09 |
| hsa-miR-3926-1 | 1.583508323 | 1.338764844 | 0.2 | 1.39E-06 |
| hsa-miR-34a | 8.84666537 | 8.602983944 | 0.2 | 1.80E-03 |
| hsa-miR-23a | 11.84102993 | 11.59998766 | 0.2 | 1.39E-02 |
| hsa-miR-20a | 7.926731282 | 7.685701238 | 0.2 | 1.39E-05 |
| hsa-miR-432 | 1.297113447 | 1.059018637 | 0.2 | 1.23E-04 |
| hsa-miR-496 | 0.679556788 | 0.443207747 | 0.2 | 8.65E-04 |
| hsa-miR-377 | 0.858738929 | 0.628030599 | 0.2 | 4.08E-03 |
| hsa-miR-92a-1 | 8.770819332 | 8.54463926 | 0.2 | 1.76E-03 |
| hsa-miR-125b-1 | 12.01642 | 11.79140918 | 0.2 | 6.37E-03 |
| hsa-miR-29b-2 | 9.876660513 | 9.657681752 | 0.2 | 1.96E-05 |
| hsa-miR-3934 | 2.782188001 | 2.566001937 | 0.2 | 3.81E-07 |
| hsa-miR-132 | 6.890418245 | 6.674391581 | 0.2 | 1.85E-04 |
| hsa-miR-625 | 6.942280938 | 6.726692447 | 0.2 | 3.17E-03 |
| hsa-miR-574 | 6.517431766 | 6.303903218 | 0.2 | 1.11E-04 |
| hsa-miR-1271 | 1.970815364 | 1.762711037 | 0.2 | 8.15E-06 |
| hsa-miR-147b | 0.719177795 | 0.513030105 | 0.2 | 3.95E-06 |
| hsa-miR-92a-2 | 13.05288334 | 12.8504414 | 0.2 | 6.20E-04 |
| hsa-miR-29a | 14.39919002 | 14.20245101 | 0.2 | 3.17E-03 |
| hsa-miR-1245 | 0.659701925 | 0.47655876 | 0.2 | 5.39E-04 |
| hsa-miR-181a-1 | 12.50244407 | 12.32314258 | 0.2 | 1.96E-02 |
| hsa-miR-17 | 9.256508741 | 9.07752643 | 0.2 | 2.50E-02 |
| hsa-miR-19b-2 | 7.014032559 | 6.836241361 | 0.2 | 5.75E-04 |
| hsa-miR-323 | 0.639194049 | 0.464135874 | 0.2 | 6.93E-03 |
| hsa-miR-506 | 0.764120933 | 0.589268465 | 0.2 | 1.22E-03 |
| hsa-miR-655 | 0.606351757 | 0.43378738 | 0.2 | 1.41E-05 |
| hsa-miR-513c | 0.417551289 | 0.27117388 | 0.1 | 1.35E-02 |
| hsa-miR-487a | 0.438473176 | 0.29384894 | 0.1 | 3.77E-05 |
| hsa-miR-659 | 1.675754891 | 1.532368425 | 0.1 | 3.97E-06 |
| hsa-miR-2277 | 0.935889327 | 0.796946663 | 0.1 | 2.48E-02 |
| hsa-miR-1262 | 0.694064977 | 0.558755071 | 0.1 | 7.29E-03 |
| hsa-miR-376b | 0.4942699 | 0.367395577 | 0.1 | 2.71E-02 |
| hsa-miR-3136 | 0.434635356 | 0.309140076 | 0.1 | 1.14E-03 |
| hsa-miR-514b | 0.368075202 | 0.242615302 | 0.1 | 3.34E-03 |
| hsa-miR-433 | 0.478746275 | 0.35517433 | 0.1 | 6.26E-03 |
| hsa-miR-376a-1 | 0.413733836 | 0.291658499 | 0.1 | 2.27E-02 |
| hsa-miR-196a-2 | 0.271493484 | 0.15431544 | 0.1 | 1.66E-07 |
| hsa-miR-543 | 0.376441294 | 0.259368305 | 0.1 | 2.06E-02 |
| hsa-miR-1537 | 0.407099967 | 0.300873052 | 0.1 | 3.51E-02 |
| hsa-miR-3191 | 0.346283277 | 0.240426933 | 0.1 | 8.06E-03 |
| hsa-miR-2116 | 0.67618495 | 0.572561486 | 0.1 | 6.65E-03 |
| hsa-miR-3926-2 | 0.401143854 | 0.303819671 | 0.1 | 5.63E-03 |
| hsa-miR-656 | 0.259962174 | 0.16265151 | 0.1 | 3.42E-02 |
| hsa-miR-380 | 0.311483281 | 0.214468532 | 0.1 | 1.50E-03 |
| hsa-miR-329-2 | 0.177207626 | 0.112288154 | 0.1 | 5.71E-05 |
| hsa-miR-513b | 0.129276672 | 0.07441093 | 0.1 | 1.30E-02 |
| hsa-miR-663b | 0.016141061 | 0.000992171 | 0.02 | 4.00E-02 |
| hsa-miR-3650 | 0.015640754 | 0.040698173 | -0.03 | 5.12E-04 |
| hsa-miR-670 | 0.011907786 | 0.039104862 | -0.03 | 7.42E-03 |
| hsa-miR-124-3 | 0.019942638 | 0.05189699 | -0.03 | 1.27E-02 |
| hsa-miR-3662 | 0.078735667 | 0.13087324 | -0.1 | 5.86E-04 |
| hsa-miR-3920 | 0.338211822 | 0.426705535 | -0.1 | 1.48E-02 |
| hsa-miR-2117 | 0.234782995 | 0.333929455 | -0.1 | 6.26E-03 |
| hsa-miR-765 | 0.331396412 | 0.443670171 | -0.1 | 1.35E-03 |
| hsa-miR-488 | 0.10377774 | 0.220796234 | -0.1 | 2.75E-02 |
| hsa-miR-1291 | 0.610764347 | 0.731313095 | -0.1 | 4.59E-02 |
| hsa-miR-1250 | 0.256821863 | 0.380195695 | -0.1 | 2.04E-03 |
| hsa-miR-3664 | 0.190699018 | 0.318370513 | -0.1 | 6.93E-03 |
| hsa-miR-128-1 | 6.321842405 | 6.452845849 | -0.1 | 1.39E-03 |
| hsa-miR-374b | 6.398994937 | 6.538808229 | -0.1 | 1.24E-02 |
| hsa-miR-106a | 2.773230534 | 2.919775984 | -0.1 | 1.23E-04 |
| hsa-miR-1288 | 0.54134208 | 0.699843267 | -0.2 | 4.66E-03 |
| hsa-miR-552 | 0.386223371 | 0.550283984 | -0.2 | 4.60E-04 |
| hsa-miR-651 | 2.025014891 | 2.196516824 | -0.2 | 3.00E-02 |
| hsa-miR-3912 | 1.517633203 | 1.692850852 | -0.2 | 8.48E-04 |
| hsa-miR-940 | 1.298938315 | 1.481911483 | -0.2 | 4.69E-04 |
| hsa-miR-1468 | 3.741585311 | 3.933555959 | -0.2 | 4.44E-05 |
| hsa-miR-3193 | 0.692526307 | 0.885828249 | -0.2 | 2.32E-04 |
| hsa-miR-185 | 6.536176647 | 6.73001067 | -0.2 | 1.24E-06 |
| hsa-miR-219-1 | 2.084230802 | 2.280440961 | -0.2 | 3.05E-08 |
| hsa-miR-30c-1 | 1.808222279 | 2.007834357 | -0.2 | 3.38E-02 |
| hsa-miR-3652 | 0.950806078 | 1.153746706 | -0.2 | 4.92E-03 |
| hsa-miR-888 | 0.180065078 | 0.387237022 | -0.2 | 5.71E-05 |
| hsa-miR-22 | 16.37855892 | 16.58956517 | -0.2 | 2.50E-05 |
| hsa-miR-1287 | 5.119684282 | 5.332924536 | -0.2 | 1.47E-05 |
| hsa-miR-95 | 3.725793675 | 3.951836715 | -0.2 | 2.22E-02 |
| hsa-miR-628 | 4.221924052 | 4.448332659 | -0.2 | 1.67E-04 |
| hsa-miR-598 | 6.50567138 | 6.734868288 | -0.2 | 3.00E-02 |
| hsa-miR-195 | 4.759038705 | 4.995335636 | -0.2 | 1.39E-02 |
| hsa-miR-96 | 3.765123654 | 4.01327777 | -0.2 | 3.97E-06 |
| hsa-miR-30e | 14.17889552 | 14.43976216 | -0.3 | 2.07E-02 |
| hsa-miR-876 | 0.125222485 | 0.387825727 | -0.3 | 9.85E-04 |
| hsa-miR-935 | 1.088702892 | 1.356112762 | -0.3 | 2.02E-05 |
| hsa-miR-98 | 6.086680758 | 6.356746701 | -0.3 | 1.35E-03 |
| hsa-miR-1275 | 0.640910767 | 0.912570458 | -0.3 | 8.69E-03 |
| hsa-miR-584 | 5.80239172 | 6.074416643 | -0.3 | 6.35E-03 |
| hsa-miR-148b | 7.994141279 | 8.272978158 | -0.3 | 4.60E-04 |
| hsa-miR-30a | 16.10657965 | 16.39786579 | -0.3 | 3.51E-02 |
| hsa-miR-30c-2 | 10.20439166 | 10.51053941 | -0.3 | 5.20E-06 |
| hsa-miR-138-1 | 4.443090918 | 4.753960385 | -0.3 | 4.78E-03 |
| hsa-miR-143 | 15.80693097 | 16.11967782 | -0.3 | 9.90E-03 |
| hsa-miR-20b | 3.019466569 | 3.340248592 | -0.3 | 1.15E-02 |
| hsa-miR-126 | 12.22317163 | 12.55766264 | -0.3 | 5.12E-03 |
| hsa-miR-892a | 0.319228578 | 0.665907868 | -0.3 | 2.90E-02 |
| hsa-miR-301b | 0.615890629 | 0.962907275 | -0.3 | 3.14E-02 |
| hsa-miR-1180 | 5.301228683 | 5.650341814 | -0.3 | 1.36E-03 |
| hsa-miR-542 | 9.727588809 | 10.07733279 | -0.3 | 1.02E-05 |
| hsa-miR-182 | 13.1921066 | 13.54693531 | -0.4 | 8.73E-10 |
| hsa-miR-338 | 8.653818497 | 9.01007118 | -0.4 | 1.68E-05 |
| hsa-miR-148a | 14.82444323 | 15.1908082 | -0.4 | 6.38E-03 |
| hsa-miR-652 | 4.627738585 | 5.014189907 | -0.4 | 1.53E-03 |
| hsa-miR-190 | 2.780916965 | 3.175975723 | -0.4 | 1.29E-02 |
| hsa-miR-1251 | 6.837326176 | 7.270191876 | -0.4 | 2.59E-07 |
| hsa-miR-363 | 2.151802924 | 2.584879986 | -0.4 | 1.39E-05 |
| hsa-miR-130b | 3.824012065 | 4.261431696 | -0.4 | 4.57E-07 |
| hsa-miR-183 | 11.54204783 | 12.00673562 | -0.5 | 3.24E-02 |
| hsa-miR-3074 | 1.975674704 | 2.455549723 | -0.5 | 1.10E-03 |
| hsa-miR-873 | 0.345394418 | 0.830134943 | -0.5 | 6.17E-03 |
| hsa-miR-139 | 6.807554033 | 7.301355032 | -0.5 | 1.07E-02 |
| hsa-miR-676 | 1.441282989 | 1.939381454 | -0.5 | 7.10E-04 |
| hsa-miR-152 | 8.027059459 | 8.53274782 | -0.5 | 2.11E-05 |
| hsa-miR-874 | 6.190229756 | 6.717858836 | -0.5 | 2.59E-07 |
| hsa-miR-577 | 1.664544516 | 2.225827711 | -0.6 | 1.38E-03 |
| hsa-miR-345 | 5.445523843 | 6.019934521 | -0.6 | 2.75E-02 |
| hsa-miR-7-3 | 0.621607284 | 1.211471361 | -0.6 | 1.01E-04 |
| hsa-miR-1179 | 1.228823678 | 2.134749714 | -0.9 | 1.16E-02 |
| hsa-miR-7-2 | 1.492473349 | 2.630513125 | -1.1 | 3.00E-02 |
| hsa-miR-204 | 3.335985012 | 4.684290638 | -1.3 | 2.11E-05 |

Student's T-test with BH corrected p value ≤0.05.

BH: Benjamini–Hochberg.
