# Supplementary material for: Integrated microRNA, gene expression and transcription factors signature in papillary thyroid cancer with lymph node metastasis
Source: PeerJ. 2016 Jun 15;4:e2119. doi: 10.7717/peerj.2119 (PMC4918724; doi:10.7717/peerj.2119)
Supplement: Table S3 [file peerj-04-2119-s003.docx]

Supplementary Table 3. 78 significantly deregulated miRNAs with respective log_2_ fold change and p value in PTCs (LNM-P and LNN) versus normal thyroid.

|  | **PTC LNM-P vs normal thyroid** | | **PTC LNN vs normal thyroid** | |
| --- | --- | --- | --- | --- |
| **miRNA ID** | **Log2 fold change** | **BH adj. p value** | **Log2 fold change** | **BH adj. p value** |
| hsa-miR-146b | 5.7 | 1.95E-06 | 4.0 | 4.56E-04 |
| hsa-miR-551b | 3.5 | 2.00E-06 | 2.6 | 4.56E-04 |
| hsa-miR-221 | 3.2 | 1.91E-06 | 2.4 | 4.47E-04 |
| hsa-miR-375 | 3.1 | 1.18E-14 | 1.7 | 1.07E-04 |
| hsa-miR-222 | 2.9 | 4.81E-20 | 2.1 | 7.31E-13 |
| hsa-miR-34a | 2.0 | 1.84E-04 | 1.8 | 3.39E-13 |
| hsa-miR-31 | 2.0 | 9.34E-04 | ns | |
| hsa-miR-21 | 1.8 | 1.77E-02 | 1.1 | 2.70E-23 |
| hsa-miR-181b-2 | 1.7 | 5.61E-07 | 1.5 | 1.34E-02 |
| hsa-miR-187 | 1.4 | 2.22E-07 | 1.2 | 2.77E-05 |
| hsa-miR-181a-2 | 1.3 | 6.01E-25 | 1.1 | 6.17E-05 |
| hsa-miR-508 | 1.3 | 6.75E-04 | ns | |
| hsa-miR-181b-1 | 1.3 | 2.09E-07 | 1.1 | 6.31E-07 |
| hsa-miR-514-2 | 1.2 | 1.28E-03 | ns | |
| hsa-miR-934 | 1.2 | 5.07E-04 | ns | |
| hsa-miR-514-3 | 1.2 | 1.31E-03 | ns | |
| hsa-miR-514-1 | 1.2 | 3.55E-14 | ns | |
| hsa-miR-181a-1 | 1.1 | 9.53E-10 | ns | |
| hsa-miR-509-3 | 1.1 | 1.31E-03 | ns | |
| hsa-miR-509-1 | 1.1 | 3.82E-12 | ns | |
| hsa-miR-509-2 | 1.0 | 3.92E-07 | ns | |
| hsa-miR-133a-1 | -1.0 | 1.31E-02 | -1.2 | 1.41E-03 |
| hsa-miR-874 | -1.0 | 3.08E-12 | ns | |
| hsa-miR-381 | -1.0 | 9.03E-04 | -1.6 | 2.53E-03 |
| hsa-miR-365-2 | -1.0 | 8.26E-03 | ns | |
| hsa-miR-497 | -1.1 | 2.03E-02 | ns | |
| hsa-miR-365-1 | -1.1 | 7.30E-03 | ns | |
| hsa-miR-199a-1 | -1.1 | 3.56E-07 | -1.6 | 1.49E-02 |
| hsa-miR-337 | -1.1 | 5.67E-29 | -1.6 | 9.75E-30 |
| hsa-miR-1-2 | -1.1 | 1.02E-09 | -1.0 | 2.31E-10 |
| hsa-miR-214 | -1.1 | 4.36E-05 | -1.6 | 3.82E-02 |
| hsa-miR-130b | -1.1 | 1.77E-05 | ns | |
| hsa-miR-199a-2 | -1.1 | 3.82E-10 | -1.6 | 5.76E-08 |
| hsa-miR-100 | -1.2 | 3.02E-05 | -1.2 | 3.26E-07 |
| hsa-miR-193b | -1.2 | 8.56E-03 | -1.2 | 7.09E-17 |
| hsa-miR-150 | -1.2 | 3.21E-06 | -1.5 | 1.42E-02 |
| hsa-miR-199b | -1.3 | 2.64E-03 | -1.8 | 3.69E-04 |
| hsa-miR-1258 | -1.3 | 5.18E-11 | -1.3 | 2.92E-21 |
| hsa-miR-206 | -1.3 | 1.76E-15 | -1.5 | 1.98E-05 |
| hsa-miR-139 | -1.3 | 1.34E-06 | ns | |
| hsa-miR-153-2 | -1.3 | 1.44E-35 | -1.2 | 5.29E-03 |
| hsa-miR-345 | -1.4 | 2.74E-17 | ns | |
| hsa-miR-675 | -1.4 | 3.75E-15 | -1.9 | 2.17E-02 |
| hsa-miR-190 | -1.4 | 1.33E-05 | -1.0 | 4.79E-02 |
| hsa-miR-379 | -1.5 | 2.08E-05 | -2.0 | 2.43E-03 |
| hsa-miR-152 | -1.5 | 4.82E-02 | ns | |
| hsa-miR-195 | -1.5 | 2.74E-05 | -1.3 | 1.93E-19 |
| hsa-miR-3687 | -1.5 | 3.03E-03 | -1.5 | 3.40E-02 |
| hsa-miR-577 | -1.5 | 4.05E-27 | ns | |
| hsa-miR-20b | -1.6 | 3.28E-20 | -1.3 | 1.19E-04 |
| hsa-miR-3074 | -1.6 | 2.38E-24 | -1.1 | 2.49E-02 |
| hsa-miR-652 | -1.6 | 4.23E-04 | -1.2 | 5.18E-03 |
| hsa-miR-138-2 | -1.8 | 2.03E-03 | -1.6 | 6.33E-31 |
| hsa-miR-363 | -1.8 | 3.35E-30 | -1.4 | 1.75E-04 |
| hsa-miR-138-1 | -1.9 | 9.62E-29 | -1.6 | 3.03E-02 |
| hsa-miR-873 | -2.0 | 1.38E-08 | -1.5 | 1.13E-20 |
| hsa-miR-7-3 | -2.1 | 3.51E-06 | -1.5 | 6.72E-17 |
| hsa-miR-1247 | -2.1 | 1.57E-38 | -2.6 | 2.71E-03 |
| hsa-miR-9-2 | -2.2 | 9.67E-07 | -2.3 | 2.44E-16 |
| hsa-miR-9-1 | -2.2 | 1.39E-05 | -2.3 | 6.00E-05 |
| hsa-miR-451 | -2.7 | 5.72E-14 | -2.6 | 5.73E-15 |
| hsa-miR-144 | -2.7 | 2.54E-07 | -2.5 | 4.75E-12 |
| hsa-miR-486 | -2.8 | 1.25E-10 | -2.6 | 1.88E-02 |
| hsa-miR-1179 | -3.1 | 2.28E-07 | -2.2 | 4.73E-07 |
| hsa-miR-7-2 | -3.2 | 1.34E-12 | -2.0 | 4.52E-16 |
| hsa-miR-204 | -3.6 | 3.00E-18 | -2.3 | 1.40E-02 |
| hsa-miR-218-2 | ns | | -1.0 | 2.21E-03 |
| hsa-miR-758 | ns | | -1.0 | 3.43E-21 |
| hsa-miR-18a | ns | | -1.0 | 3.16E-19 |
| hsa-miR-142 | ns | | -1.1 | 3.56E-08 |
| hsa-miR-942 | ns | | -1.1 | 4.94E-06 |
| hsa-miR-411 | ns | | -1.3 | 7.14E-11 |
| hsa-miR-223 | ns | | -1.3 | 2.34E-05 |
| hsa-miR-136 | ns | | -1.4 | 1.68E-14 |
| hsa-miR-654 | ns | | -1.4 | 7.79E-06 |
| hsa-miR-708 | ns | | -1.4 | 2.67E-03 |
| hsa-miR-127 | ns | | -1.6 | 5.82E-11 |
| hsa-miR-134 | ns | | -1.6 | 4.96E-02 |

Student's T-test with BH corrected p value ≤0.05; log_2_ fold change ≥ 1 or ≤ -1.

ns: not significant

BH: Benjamini–Hochberg
