# Supplementary material for: Integrated microRNA, gene expression and transcription factors signature in papillary thyroid cancer with lymph node metastasis
Source: PeerJ. 2016 Jun 15;4:e2119. doi: 10.7717/peerj.2119 (PMC4918724; doi:10.7717/peerj.2119)
Supplement: Table S4 [file peerj-04-2119-s005.docx]

Supplementary Table 4. 407 significantly deregulated genes with respective log_2_ fold change and p value in PTC LNM-P versus PTC LNN.

| **Gene ID** | **Mean expression in PTC LNM-P (Log_2_ normalized)** | **Mean expression in PTC LNN (Log_2_ normalized)** | **Log_2_ fold change** | **BH adj. p value** |
| --- | --- | --- | --- | --- |
| SFTPB | 13.61530051 | 10.50643894 | 3.1 | 4.89E-14 |
| CLDN10 | 8.019179374 | 5.140256301 | 2.9 | 9.30E-14 |
| SYT12 | 10.35573883 | 7.52954228 | 2.8 | 1.68E-13 |
| TM7SF4 | 10.15923495 | 7.333978581 | 2.8 | 4.77E-11 |
| TMPRSS4 | 9.933884789 | 7.298949639 | 2.6 | 5.48E-14 |
| TMPRSS6 | 9.39017764 | 6.792407543 | 2.6 | 2.14E-11 |
| MUC21 | 7.882964439 | 5.365354851 | 2.5 | 4.47E-13 |
| B3GNT3 | 9.324425222 | 6.818579459 | 2.5 | 3.21E-13 |
| SLC34A2 | 15.47901109 | 13.04702091 | 2.4 | 5.52E-13 |
| KLK7 | 8.644829703 | 6.235904009 | 2.4 | 1.75E-09 |
| CHI3L1 | 12.22090214 | 9.813591076 | 2.4 | 2.04E-12 |
| ST6GALNAC5 | 7.488427745 | 5.096140162 | 2.4 | 6.47E-13 |
| SLC27A6 | 9.983631767 | 7.624172037 | 2.4 | 1.97E-11 |
| FN1 | 17.9620776 | 15.71231387 | 2.2 | 3.45E-14 |
| CEACAM6 | 6.984634698 | 4.767403246 | 2.2 | 6.71E-11 |
| TACSTD2 | 12.92723325 | 10.72604781 | 2.2 | 4.79E-13 |
| KCNN4 | 9.768476697 | 7.638533351 | 2.1 | 5.48E-14 |
| TMEM92 | 6.132705704 | 4.00926407 | 2.1 | 7.62E-13 |
| KLK10 | 10.24040334 | 8.144012925 | 2.1 | 1.18E-08 |
| CST6 | 10.92469474 | 8.86740555 | 2.1 | 1.26E-11 |
| IVL | 5.845167903 | 3.798545831 | 2.0 | 5.93E-11 |
| CXCL14 | 10.98264765 | 8.996581777 | 2.0 | 6.15E-07 |
| COL11A1 | 5.039414133 | 3.065642641 | 2.0 | 4.37E-08 |
| ADCY8 | 6.086321324 | 4.114714343 | 2.0 | 6.42E-09 |
| SFN | 8.791302738 | 6.864113347 | 1.9 | 8.56E-12 |
| PDZK1IP1 | 9.603863523 | 7.686034685 | 1.9 | 3.89E-10 |
| LOC400794 | 6.934851131 | 5.020539463 | 1.9 | 5.38E-11 |
| SYTL5 | 8.794794332 | 6.898248721 | 1.9 | 7.99E-11 |
| C2CD4A | 6.62163385 | 4.725932648 | 1.9 | 4.19E-11 |
| ALOX15B | 9.866673812 | 7.993460222 | 1.9 | 2.05E-12 |
| IGFL2 | 6.068333258 | 4.204367421 | 1.9 | 1.75E-09 |
| SYT1 | 6.659662962 | 4.808544965 | 1.9 | 4.15E-12 |
| SFRP2 | 8.952371682 | 7.113252749 | 1.8 | 1.37E-06 |
| CXCL17 | 7.664840949 | 5.848033576 | 1.8 | 1.32E-10 |
| TMPRSS11E | 4.648145887 | 2.833072721 | 1.8 | 8.29E-10 |
| RASGRF1 | 7.670969043 | 5.857082309 | 1.8 | 2.07E-11 |
| KLK11 | 6.766058359 | 4.962905574 | 1.8 | 2.28E-05 |
| ALOX5 | 10.33279399 | 8.554785804 | 1.8 | 5.25E-12 |
| CLDN16 | 9.012398218 | 7.253506922 | 1.8 | 5.72E-10 |
| C1orf106 | 7.040913071 | 5.28245016 | 1.8 | 1.73E-11 |
| MMP7 | 6.916723978 | 5.158397334 | 1.8 | 9.36E-08 |
| ESPN | 6.670423664 | 4.913805189 | 1.8 | 2.46E-10 |
| IL1RL1 | 7.492893305 | 5.742344514 | 1.8 | 2.64E-07 |
| DSC3 | 6.096826049 | 4.370615742 | 1.7 | 2.30E-08 |
| NGEF | 9.736028569 | 8.010135161 | 1.7 | 8.84E-12 |
| VGLL1 | 4.407929242 | 2.69065627 | 1.7 | 9.02E-11 |
| LAMB3 | 11.75606597 | 10.04836573 | 1.7 | 1.02E-11 |
| GABRB2 | 10.81835042 | 9.112334427 | 1.7 | 9.78E-12 |
| CCL17 | 5.300229655 | 3.594745322 | 1.7 | 1.38E-10 |
| SERPINA1 | 15.15897102 | 13.45655585 | 1.7 | 8.84E-12 |
| COMP | 8.750426531 | 7.048574258 | 1.7 | 4.41E-07 |
| NMU | 6.677532292 | 4.980347568 | 1.7 | 8.51E-07 |
| PSG8 | 4.186717299 | 2.489855287 | 1.7 | 2.12E-07 |
| GAP43 | 6.097041703 | 4.409177603 | 1.7 | 6.17E-08 |
| MYBPH | 4.603043124 | 2.92374587 | 1.7 | 7.74E-12 |
| PDLIM4 | 11.33254609 | 9.679060248 | 1.7 | 1.85E-12 |
| KRT15 | 6.738512537 | 5.087623288 | 1.7 | 1.05E-11 |
| TM4SF4 | 5.894551557 | 4.250342868 | 1.6 | 1.27E-09 |
| CD1A | 5.680392818 | 4.038585552 | 1.6 | 7.08E-08 |
| CACNG4 | 6.214257123 | 4.577579737 | 1.6 | 8.26E-09 |
| COL10A1 | 6.390949841 | 4.763600851 | 1.6 | 3.01E-08 |
| SLPI | 10.39069157 | 8.775798539 | 1.6 | 1.76E-09 |
| S100B | 6.575140878 | 4.962930787 | 1.6 | 1.54E-09 |
| EREG | 4.17899968 | 2.575403438 | 1.6 | 2.46E-09 |
| SCEL | 10.50903378 | 8.910120513 | 1.6 | 4.89E-13 |
| KLK6 | 4.956683283 | 3.359202172 | 1.6 | 1.29E-06 |
| LIPH | 9.619505315 | 8.036141781 | 1.6 | 9.79E-11 |
| FRMD5 | 6.189587212 | 4.607368459 | 1.6 | 5.38E-10 |
| STAC | 6.776425723 | 5.201356502 | 1.6 | 6.86E-12 |
| WNT10A | 6.997639788 | 5.429349473 | 1.6 | 2.28E-08 |
| CD207 | 5.930253523 | 4.366840907 | 1.6 | 3.07E-08 |
| PVRL4 | 9.310188373 | 7.75290352 | 1.6 | 5.76E-12 |
| LCN2 | 9.004487445 | 7.449769106 | 1.6 | 1.00E-07 |
| MYO1G | 9.35135179 | 7.806694545 | 1.5 | 1.88E-11 |
| SIGLEC6 | 4.89974274 | 3.358154912 | 1.5 | 3.34E-06 |
| ZCCHC12 | 12.69715385 | 11.15637642 | 1.5 | 1.14E-06 |
| ICAM5 | 8.430907518 | 6.893956886 | 1.5 | 7.39E-11 |
| ADAMTS14 | 6.207979162 | 4.67167115 | 1.5 | 1.52E-11 |
| ACTBL2 | 4.442170148 | 2.90659439 | 1.5 | 5.52E-13 |
| STRA6 | 8.646000436 | 7.115233825 | 1.5 | 4.32E-08 |
| KRT19 | 12.67061607 | 11.14031965 | 1.5 | 1.18E-11 |
| SLC6A20 | 6.014231738 | 4.486168649 | 1.5 | 5.76E-07 |
| CBLN1 | 7.790519724 | 6.263720306 | 1.5 | 2.58E-09 |
| CATSPER1 | 5.158877177 | 3.639727365 | 1.5 | 1.13E-11 |
| DPP4 | 11.65662202 | 10.13971136 | 1.5 | 1.08E-10 |
| VTCN1 | 5.095249274 | 3.59369187 | 1.5 | 3.23E-06 |
| PSG1 | 4.807725263 | 3.315428367 | 1.5 | 1.33E-07 |
| PLAC2 | 5.210883235 | 3.72272154 | 1.5 | 8.96E-10 |
| CCL13 | 6.003174612 | 4.515691504 | 1.5 | 1.12E-09 |
| CRLF1 | 9.636567522 | 8.150665661 | 1.5 | 9.12E-06 |
| CCL20 | 4.66209019 | 3.177218199 | 1.5 | 2.96E-10 |
| ELFN2 | 5.398405361 | 3.916654167 | 1.5 | 4.10E-07 |
| CYP1B1 | 10.69732761 | 9.218201346 | 1.5 | 2.25E-10 |
| KRT17 | 7.322936317 | 5.857026394 | 1.5 | 7.80E-06 |
| PRSS1 | 5.077647537 | 3.627495366 | 1.5 | 2.07E-06 |
| CSF2 | 4.34369618 | 2.895814347 | 1.4 | 2.46E-12 |
| BIRC7 | 5.731084013 | 4.289233019 | 1.4 | 4.94E-07 |
| NAPSA | 7.364253253 | 5.934890977 | 1.4 | 4.32E-08 |
| MMP13 | 2.981731596 | 1.553033393 | 1.4 | 2.90E-06 |
| C19orf59 | 4.346235209 | 2.919949264 | 1.4 | 1.50E-08 |
| CD1C | 5.675128042 | 4.249348774 | 1.4 | 2.07E-09 |
| CYP2S1 | 8.308604408 | 6.888728628 | 1.4 | 4.66E-10 |
| LUM | 10.17242055 | 8.7556002 | 1.4 | 4.93E-07 |
| LY6G6C | 4.751740307 | 3.338819262 | 1.4 | 4.81E-10 |
| IGFBP6 | 11.66228393 | 10.2502958 | 1.4 | 1.32E-10 |
| PNPLA5 | 4.534769074 | 3.127636994 | 1.4 | 4.37E-07 |
| CPNE4 | 5.671914758 | 4.26492942 | 1.4 | 2.47E-06 |
| CST2 | 4.358420346 | 2.952441905 | 1.4 | 2.64E-06 |
| C19orf21 | 7.082177136 | 5.676512703 | 1.4 | 4.32E-08 |
| TNC | 11.11527843 | 9.710073286 | 1.4 | 5.08E-11 |
| CFB | 11.13008752 | 9.724935099 | 1.4 | 5.53E-08 |
| PPP1R1B | 7.475257287 | 6.074155991 | 1.4 | 1.43E-07 |
| CADM3 | 6.918026109 | 5.517940108 | 1.4 | 4.52E-06 |
| CCL18 | 8.359525325 | 6.96034705 | 1.4 | 8.12E-07 |
| FAM178B | 5.452721786 | 4.058104588 | 1.4 | 1.73E-11 |
| CCL22 | 6.099969183 | 4.705488687 | 1.4 | 4.98E-09 |
| PTGS2 | 7.935674381 | 6.543910814 | 1.4 | 3.64E-13 |
| SDR16C5 | 4.009088889 | 2.621479002 | 1.4 | 1.49E-08 |
| MARCO | 7.239837286 | 5.852551525 | 1.4 | 1.67E-06 |
| MUC1 | 11.05535545 | 9.668605096 | 1.4 | 1.08E-09 |
| CYSLTR2 | 8.01662218 | 6.632548128 | 1.4 | 8.78E-09 |
| CRLF2 | 3.181475809 | 1.797665336 | 1.4 | 3.41E-10 |
| AHNAK2 | 10.87822895 | 9.497486467 | 1.4 | 1.02E-11 |
| CD1E | 5.277112882 | 3.905331033 | 1.4 | 1.95E-08 |
| FGFBP1 | 5.900994937 | 4.530963414 | 1.4 | 2.37E-06 |
| CD177 | 4.379961607 | 3.010456648 | 1.4 | 4.40E-05 |
| CCNA1 | 5.31255233 | 3.944161359 | 1.4 | 5.55E-11 |
| TMEM163 | 8.447280259 | 7.080277895 | 1.4 | 1.61E-08 |
| IGFN1 | 7.80740793 | 6.442050575 | 1.4 | 7.42E-04 |
| GRHL3 | 6.790883208 | 5.427108712 | 1.4 | 5.82E-11 |
| C20orf103 | 7.470030086 | 6.111464691 | 1.4 | 1.95E-06 |
| CTSE | 8.64634396 | 7.289632418 | 1.4 | 4.49E-07 |
| GALNT5 | 4.902522722 | 3.548359377 | 1.4 | 4.43E-09 |
| MACC1 | 8.971202522 | 7.617701395 | 1.4 | 3.47E-12 |
| ARSI | 4.192110821 | 2.838719247 | 1.4 | 6.29E-10 |
| LRG1 | 7.8780661 | 6.531438843 | 1.3 | 1.08E-10 |
| HMGA2 | 9.684922087 | 8.340533489 | 1.3 | 4.44E-08 |
| FXYD3 | 4.508921848 | 3.165722587 | 1.3 | 1.50E-05 |
| KCNQ3 | 6.676279717 | 5.338719878 | 1.3 | 8.27E-12 |
| COL8A2 | 11.39463518 | 10.05812256 | 1.3 | 4.89E-13 |
| PLAU | 11.64633118 | 10.31658618 | 1.3 | 6.80E-12 |
| BNC1 | 5.637255917 | 4.307734675 | 1.3 | 9.69E-08 |
| IL8 | 6.959943359 | 5.640209642 | 1.3 | 1.34E-09 |
| ARHGEF38 | 4.008576084 | 2.689296943 | 1.3 | 4.09E-10 |
| COL7A1 | 6.570448833 | 5.257404514 | 1.3 | 1.88E-07 |
| CXCL2 | 7.722735326 | 6.41206011 | 1.3 | 2.30E-08 |
| KRT13 | 4.10686979 | 2.798029022 | 1.3 | 4.02E-08 |
| CXCL5 | 3.462011767 | 2.15797286 | 1.3 | 1.98E-09 |
| TRY6 | 3.610879756 | 2.307692118 | 1.3 | 5.01E-07 |
| FAM5B | 4.807061208 | 3.506538659 | 1.3 | 5.57E-08 |
| CREB5 | 8.185219909 | 6.889085725 | 1.3 | 1.06E-14 |
| TNFSF11 | 3.828170946 | 2.533881806 | 1.3 | 9.01E-10 |
| FAM176A | 8.476489089 | 7.182362818 | 1.3 | 5.81E-11 |
| C19orf33 | 9.621427659 | 8.329237613 | 1.3 | 1.22E-09 |
| SLC18A3 | 3.013560974 | 1.724649174 | 1.3 | 3.90E-06 |
| PKP2 | 6.47796077 | 5.192125363 | 1.3 | 5.47E-09 |
| GJB3 | 8.408247313 | 7.126523443 | 1.3 | 1.95E-08 |
| GPR115 | 3.490846212 | 2.209392377 | 1.3 | 4.20E-08 |
| TMEM100 | 7.565853854 | 6.290519082 | 1.3 | 4.22E-08 |
| CST5 | 3.589575763 | 2.316308255 | 1.3 | 4.20E-08 |
| COL1A1 | 13.4434538 | 12.17127321 | 1.3 | 1.41E-08 |
| LOX | 8.511246787 | 7.249549754 | 1.3 | 2.03E-11 |
| FCER1A | 6.123820638 | 4.864206142 | 1.3 | 6.26E-09 |
| RSPO4 | 8.34650185 | 7.088646356 | 1.3 | 6.63E-09 |
| KRT6A | 3.865818098 | 2.608367554 | 1.3 | 8.74E-05 |
| TRIM29 | 7.761450446 | 6.505995058 | 1.3 | 9.23E-06 |
| CDH11 | 8.739196536 | 7.4857847 | 1.3 | 3.18E-08 |
| MUC16 | 5.243925746 | 3.997817715 | 1.2 | 2.39E-04 |
| C10orf55 | 5.662613636 | 4.417252748 | 1.2 | 7.62E-13 |
| DMBX1 | 4.413932736 | 3.171679024 | 1.2 | 3.24E-05 |
| TP63 | 5.852735234 | 4.613535553 | 1.2 | 1.84E-08 |
| FAM20A | 10.79469862 | 9.561099934 | 1.2 | 1.17E-09 |
| F5 | 6.925601744 | 5.692330595 | 1.2 | 3.90E-06 |
| NFE2L3 | 9.602855701 | 8.369623848 | 1.2 | 1.24E-10 |
| SIGLEC15 | 4.721714131 | 3.497376461 | 1.2 | 2.20E-08 |
| EPHB3 | 8.903072327 | 7.681235362 | 1.2 | 3.79E-12 |
| FUT3 | 4.547011384 | 3.327079454 | 1.2 | 5.75E-08 |
| FOXQ1 | 10.04907432 | 8.830391586 | 1.2 | 4.78E-10 |
| FLJ42709 | 8.189266657 | 6.97161858 | 1.2 | 5.25E-12 |
| NELL2 | 11.46346983 | 10.2502949 | 1.2 | 6.23E-07 |
| PROS1 | 12.74866893 | 11.53706693 | 1.2 | 3.69E-13 |
| ITGB8 | 9.3478091 | 8.142542203 | 1.2 | 1.95E-10 |
| PHACTR3 | 4.449961615 | 3.244778896 | 1.2 | 1.58E-08 |
| EHF | 7.795304293 | 6.594490273 | 1.2 | 1.04E-08 |
| RUNX2 | 8.592166873 | 7.392807993 | 1.2 | 2.80E-11 |
| CLDN1 | 13.27575819 | 12.07819036 | 1.2 | 5.03E-10 |
| ARSJ | 6.585230982 | 5.388528422 | 1.2 | 1.73E-11 |
| RXRG | 10.55007947 | 9.3560602 | 1.2 | 3.56E-06 |
| VCAN | 9.313412539 | 8.12244824 | 1.2 | 2.99E-08 |
| ARHGAP36 | 9.089614506 | 7.89989248 | 1.2 | 1.33E-02 |
| RPS4Y1 | 4.237078059 | 3.049628101 | 1.2 | 4.86E-02 |
| LDLR | 9.299242849 | 8.11760529 | 1.2 | 1.78E-12 |
| SPOCK2 | 12.46074661 | 11.28120669 | 1.2 | 3.02E-09 |
| KRT80 | 9.160047283 | 7.981152721 | 1.2 | 5.09E-10 |
| HLA-DQB2 | 8.290071877 | 7.111862272 | 1.2 | 3.36E-07 |
| TRPC5 | 7.054718314 | 5.881126116 | 1.2 | 7.00E-04 |
| PLAUR | 9.152673705 | 7.979449734 | 1.2 | 1.14E-11 |
| MMP12 | 4.032543103 | 2.861905142 | 1.2 | 5.25E-06 |
| SPON1 | 6.583886059 | 5.414967494 | 1.2 | 1.02E-06 |
| DUSP5 | 11.84154336 | 10.67472419 | 1.2 | 3.53E-12 |
| CD70 | 4.26587527 | 3.102119566 | 1.2 | 1.02E-06 |
| TIMP1 | 13.86306781 | 12.7027367 | 1.2 | 7.54E-11 |
| LCN6 | 5.828141845 | 4.669785022 | 1.2 | 6.97E-04 |
| PDE5A | 12.6130344 | 11.45688139 | 1.2 | 7.62E-13 |
| FCHO1 | 6.591677542 | 5.437590236 | 1.2 | 4.19E-09 |
| SPTBN2 | 8.569668881 | 7.417175366 | 1.2 | 1.95E-10 |
| GLDN | 6.608976978 | 5.457345074 | 1.2 | 1.05E-04 |
| STMN2 | 4.468380546 | 3.317119133 | 1.2 | 5.58E-06 |
| PTPRE | 10.60639728 | 9.45614287 | 1.2 | 1.54E-13 |
| MMP16 | 7.215456373 | 6.065686199 | 1.1 | 9.16E-09 |
| SLC6A14 | 4.848323619 | 3.700679626 | 1.1 | 3.97E-05 |
| ELF3 | 10.38223658 | 9.23524524 | 1.1 | 3.38E-12 |
| LRRC15 | 5.190486043 | 4.045052347 | 1.1 | 6.60E-04 |
| LGALS3 | 13.03328786 | 11.88931743 | 1.1 | 1.60E-12 |
| UNC5CL | 7.304307007 | 6.160962646 | 1.1 | 1.38E-12 |
| GRM4 | 6.351895315 | 5.211380382 | 1.1 | 6.85E-05 |
| C2CD4B | 5.657130115 | 4.518572735 | 1.1 | 9.93E-08 |
| LOC554202 | 7.476577866 | 6.339500236 | 1.1 | 5.25E-06 |
| FAP | 7.09419985 | 5.958869716 | 1.1 | 4.42E-08 |
| RNF183 | 4.829261769 | 3.693983404 | 1.1 | 1.72E-07 |
| XKRX | 7.381698013 | 6.252461475 | 1.1 | 5.93E-09 |
| CHIT1 | 8.40625394 | 7.278290273 | 1.1 | 2.64E-04 |
| PLEKHA6 | 8.295987234 | 7.168494691 | 1.1 | 1.31E-09 |
| ZCCHC16 | 6.923180228 | 5.79813681 | 1.1 | 1.38E-04 |
| TNFSF9 | 4.838758893 | 3.717696889 | 1.1 | 5.06E-08 |
| PLXNC1 | 9.011438183 | 7.893650496 | 1.1 | 6.16E-08 |
| MGAT3 | 9.514411604 | 8.398111068 | 1.1 | 1.23E-07 |
| DTX4 | 13.09434304 | 11.98158448 | 1.1 | 2.10E-10 |
| SCNN1A | 11.9769459 | 10.86560834 | 1.1 | 2.49E-09 |
| PRSS3 | 3.833562688 | 2.724545972 | 1.1 | 4.88E-07 |
| TREM1 | 6.813659649 | 5.706525858 | 1.1 | 6.41E-08 |
| BEAN | 7.128557651 | 6.021735051 | 1.1 | 3.08E-11 |
| RDH5 | 8.428292617 | 7.324662353 | 1.1 | 1.41E-08 |
| HES2 | 4.980739602 | 3.877294591 | 1.1 | 2.24E-08 |
| CDSN | 7.784680787 | 6.683043255 | 1.1 | 1.76E-07 |
| CFH | 11.42652757 | 10.32852939 | 1.1 | 2.24E-10 |
| S100A2 | 8.677324089 | 7.580368421 | 1.1 | 5.25E-09 |
| ERBB3 | 10.75909586 | 9.662830349 | 1.1 | 2.93E-12 |
| CLDN2 | 7.315261368 | 6.219169464 | 1.1 | 1.32E-05 |
| S100A4 | 11.47814445 | 10.38246134 | 1.1 | 1.66E-09 |
| LPAR5 | 10.42302641 | 9.327401668 | 1.1 | 2.73E-10 |
| TCN1 | 2.470034187 | 1.375023632 | 1.1 | 5.81E-07 |
| CAMK2N1 | 11.64029846 | 10.54657269 | 1.1 | 6.91E-10 |
| MXRA8 | 12.549394 | 11.45662212 | 1.1 | 2.00E-10 |
| CITED1 | 11.74221324 | 10.6495648 | 1.1 | 3.50E-05 |
| SFRP4 | 7.813471116 | 6.721312132 | 1.1 | 2.19E-04 |
| IL1RAP | 9.596094642 | 8.504504401 | 1.1 | 1.38E-12 |
| ADAM6 | 12.41083862 | 11.32011293 | 1.1 | 7.81E-03 |
| INHBA | 5.651505718 | 4.562286539 | 1.1 | 6.79E-09 |
| SNAP25 | 6.014278028 | 4.925963943 | 1.1 | 9.28E-08 |
| TUBB3 | 9.115946385 | 8.028093029 | 1.1 | 2.84E-09 |
| ROR1 | 4.832012975 | 3.74773808 | 1.1 | 2.47E-08 |
| PRR15 | 10.55611388 | 9.472201023 | 1.1 | 4.28E-05 |
| C7 | 8.532049056 | 7.448953483 | 1.1 | 8.15E-04 |
| SPOCD1 | 5.303848063 | 4.22137726 | 1.1 | 1.00E-08 |
| OR4D10 | 2.797116921 | 1.717377117 | 1.1 | 4.28E-09 |
| LIMS3 | 4.864110058 | 3.785710736 | 1.1 | 1.73E-09 |
| S100A10 | 11.49135558 | 10.4136631 | 1.1 | 1.05E-11 |
| C1orf116 | 10.58776802 | 9.512479009 | 1.1 | 3.41E-10 |
| SLCO4A1 | 6.677534812 | 5.603416607 | 1.1 | 1.62E-09 |
| KRT16 | 2.785527409 | 1.712807536 | 1.1 | 1.81E-05 |
| CDH3 | 11.32762233 | 10.25587188 | 1.1 | 4.80E-09 |
| DHRS9 | 5.398396425 | 4.330273636 | 1.1 | 9.44E-07 |
| FAM83A | 5.699495512 | 4.632113501 | 1.1 | 1.21E-05 |
| AREG | 5.365863557 | 4.300934287 | 1.1 | 5.93E-07 |
| SDK1 | 8.757831386 | 7.695075097 | 1.1 | 7.26E-06 |
| CARD11 | 7.497693958 | 6.43507677 | 1.1 | 1.43E-07 |
| CTHRC1 | 7.552080921 | 6.49177543 | 1.1 | 9.22E-07 |
| LEMD1 | 6.346736354 | 5.28998827 | 1.1 | 1.92E-10 |
| HPCAL4 | 7.806951139 | 6.753347461 | 1.1 | 8.70E-06 |
| CH25H | 6.779124401 | 5.726589221 | 1.1 | 5.14E-08 |
| KDM5D | 3.410149079 | 2.361701153 | 1.0 | 3.97E-02 |
| SOX11 | 4.279871834 | 3.242569391 | 1.0 | 9.12E-07 |
| TMEM184A | 8.025182426 | 6.987885208 | 1.0 | 4.39E-10 |
| ICAM4 | 6.885903679 | 5.85342649 | 1.0 | 2.55E-07 |
| C13orf33 | 8.467855269 | 7.436429581 | 1.0 | 4.25E-12 |
| PLA2G5 | 5.032198946 | 4.001429067 | 1.0 | 3.02E-07 |
| EPHA10 | 7.342023804 | 6.313084788 | 1.0 | 1.20E-06 |
| SERPINA3 | 4.938102789 | 3.911634007 | 1.0 | 9.33E-05 |
| MET | 13.52373139 | 12.49730972 | 1.0 | 7.72E-14 |
| SCARA3 | 11.8469933 | 10.82107779 | 1.0 | 3.57E-09 |
| ICAM1 | 11.90746606 | 10.89216399 | 1.0 | 6.09E-13 |
| DCN | 10.29784807 | 9.283113577 | 1.0 | 1.23E-04 |
| RETN | 2.442403728 | 1.427807345 | 1.0 | 1.18E-06 |
| PRMT8 | 4.838010103 | 3.824076235 | 1.0 | 3.17E-03 |
| CDH22 | 5.960023612 | 4.946181013 | 1.0 | 2.12E-05 |
| CD1B | 2.905526456 | 1.892136963 | 1.0 | 1.30E-06 |
| XDH | 4.290125377 | 3.278682619 | 1.0 | 1.40E-04 |
| ALDH1A3 | 9.180737979 | 8.170916388 | 1.0 | 3.61E-07 |
| FLJ16779 | 4.414017016 | 3.40427011 | 1.0 | 7.06E-06 |
| PODNL1 | 4.931118045 | 3.922111082 | 1.0 | 1.30E-06 |
| KCNJ2 | 9.305029404 | 8.298316371 | 1.0 | 3.64E-08 |
| HAS1 | 2.23931266 | 1.233336418 | 1.0 | 6.46E-07 |
| PI15 | 3.189949144 | 2.184484894 | 1.0 | 9.08E-10 |
| HLA-G | 8.307714517 | 7.303027374 | 1.0 | 3.31E-06 |
| RUNX1 | 10.31951656 | 9.315481944 | 1.0 | 5.52E-13 |
| MICALCL | 5.8397551 | 4.835978746 | 1.0 | 4.98E-09 |
| KIAA1324 | 5.720022308 | 6.721360471 | -1.0 | 5.57E-05 |
| TCEAL2 | 6.181367689 | 7.190309034 | -1.0 | 1.28E-04 |
| DHDPSL | 2.990022191 | 4.000932046 | -1.0 | 5.55E-08 |
| CIB4 | 1.106640389 | 2.117638982 | -1.0 | 1.74E-07 |
| ALDH1L1 | 3.938689886 | 4.949945066 | -1.0 | 7.18E-07 |
| KCNJ13 | 3.495467034 | 4.508285645 | -1.0 | 1.74E-05 |
| MT1M | 4.799053336 | 5.820476411 | -1.0 | 2.05E-07 |
| CD36 | 7.798802443 | 8.823568929 | -1.0 | 1.54E-07 |
| SLC14A2 | 2.582204101 | 3.610816301 | -1.0 | 5.58E-05 |
| LOC389332 | 1.104408183 | 2.136879654 | -1.0 | 1.84E-08 |
| MIOX | 4.600991007 | 5.63502846 | -1.0 | 2.80E-07 |
| SH2D6 | 1.624180118 | 2.659917737 | -1.0 | 2.22E-09 |
| TBX22 | 1.973676578 | 3.009832174 | -1.0 | 8.87E-06 |
| BTBD17 | 0.715524954 | 1.752259598 | -1.0 | 7.85E-08 |
| GCK | 2.149133627 | 3.189716556 | -1.0 | 1.09E-09 |
| AGTR1 | 3.558686359 | 4.603017245 | -1.0 | 1.25E-06 |
| TFCP2L1 | 7.556885152 | 8.603743382 | -1.0 | 6.22E-08 |
| C7orf52 | 2.697020766 | 3.745768443 | -1.0 | 1.55E-05 |
| APOA1 | 3.133640309 | 4.186523199 | -1.1 | 1.45E-06 |
| TDH | 1.723583242 | 2.777677336 | -1.1 | 4.48E-10 |
| IGDCC3 | 4.161081387 | 5.215989736 | -1.1 | 9.47E-08 |
| PAX1 | 1.010205961 | 2.075552641 | -1.1 | 2.69E-06 |
| KCTD8 | 4.221910917 | 5.292295263 | -1.1 | 5.95E-09 |
| TRPV6 | 3.84581397 | 4.91732005 | -1.1 | 6.76E-08 |
| SOD3 | 8.877289786 | 9.95082292 | -1.1 | 7.39E-11 |
| OGDHL | 7.700091106 | 8.774431114 | -1.1 | 1.09E-08 |
| FAM155B | 7.865761754 | 8.940449529 | -1.1 | 1.82E-06 |
| TRPM3 | 1.323531173 | 2.401544917 | -1.1 | 3.13E-09 |
| NWD1 | 3.113029347 | 4.197211766 | -1.1 | 4.67E-06 |
| FOXJ1 | 3.708511052 | 4.79899263 | -1.1 | 1.85E-05 |
| FER1L6 | 1.238522277 | 2.331105836 | -1.1 | 6.65E-07 |
| ATP2C2 | 5.000238936 | 6.098823134 | -1.1 | 1.36E-08 |
| KIF19 | 3.111448176 | 4.213111251 | -1.1 | 5.72E-07 |
| LRRTM1 | 3.039939623 | 4.142236095 | -1.1 | 3.69E-05 |
| ADIG | 1.650472133 | 2.752856261 | -1.1 | 1.18E-09 |
| MSI1 | 3.328186959 | 4.434407827 | -1.1 | 4.95E-07 |
| LOC100132354 | 3.056546038 | 4.170256348 | -1.1 | 2.23E-07 |
| C11orf20 | 0.734204802 | 1.857896409 | -1.1 | 1.91E-11 |
| EBF4 | 7.714179311 | 8.840880515 | -1.1 | 1.54E-10 |
| GABRB3 | 6.11935967 | 7.254874749 | -1.1 | 1.37E-06 |
| OTOS | 6.219047704 | 7.358302773 | -1.1 | 1.65E-07 |
| CLCNKB | 5.855275649 | 7.003377298 | -1.1 | 2.49E-12 |
| CWH43 | 4.154977412 | 5.305198231 | -1.2 | 1.47E-05 |
| GPR98 | 4.891038382 | 6.048894738 | -1.2 | 1.11E-07 |
| SPINK5 | 3.300388901 | 4.458443008 | -1.2 | 2.29E-07 |
| BEX1 | 5.842012388 | 7.003521206 | -1.2 | 5.71E-08 |
| IGFBPL1 | 2.886166751 | 4.057917571 | -1.2 | 9.57E-09 |
| PKNOX2 | 5.561960578 | 6.735186935 | -1.2 | 1.06E-04 |
| MMEL1 | 0.99859307 | 2.178957644 | -1.2 | 1.04E-09 |
| GABRG1 | 1.793239125 | 2.973842866 | -1.2 | 1.12E-10 |
| CARTPT | 1.997543239 | 3.181919483 | -1.2 | 1.08E-03 |
| SOX3 | 1.023524932 | 2.211593334 | -1.2 | 4.89E-07 |
| NEB | 4.506901041 | 5.704715181 | -1.2 | 1.73E-06 |
| CRABP1 | 6.843231081 | 8.058429489 | -1.2 | 7.62E-05 |
| MAPK4 | 4.284239933 | 5.508667047 | -1.2 | 3.43E-09 |
| DIRAS2 | 3.715522502 | 4.949216262 | -1.2 | 1.40E-12 |
| LGI3 | 6.85579426 | 8.092945211 | -1.2 | 8.61E-11 |
| PYGM | 4.757132211 | 6.001069042 | -1.2 | 2.14E-09 |
| GRIK4 | 3.41738128 | 4.666011269 | -1.2 | 4.49E-09 |
| CNTFR | 2.780182063 | 4.042293945 | -1.3 | 4.67E-05 |
| HS6ST3 | 4.019588572 | 5.290126985 | -1.3 | 6.03E-09 |
| FAM167A | 8.587230242 | 9.861467092 | -1.3 | 2.49E-09 |
| FAM189A1 | 4.584140188 | 5.862966151 | -1.3 | 3.57E-08 |
| LINGO2 | 1.739526748 | 3.025063955 | -1.3 | 7.91E-09 |
| UPF0639 | 1.689814388 | 2.982895373 | -1.3 | 1.45E-10 |
| CAPSL | 2.90876448 | 4.202553466 | -1.3 | 2.59E-09 |
| GLDC | 3.187010854 | 4.492521087 | -1.3 | 7.18E-07 |
| C12orf39 | 2.270817992 | 3.579313884 | -1.3 | 5.60E-11 |
| KCNK16 | 1.164980746 | 2.48953894 | -1.3 | 7.87E-09 |
| BMP8A | 7.57996153 | 8.910717586 | -1.3 | 2.43E-06 |
| MPPED2 | 5.951992687 | 7.308529037 | -1.4 | 4.32E-10 |
| CDH16 | 4.979920331 | 6.340761578 | -1.4 | 5.24E-06 |
| IRX6 | 1.791412577 | 3.165799593 | -1.4 | 2.24E-08 |
| KCNAB1 | 7.650452891 | 9.025152504 | -1.4 | 5.01E-10 |
| GRIN2C | 6.043621084 | 7.425287961 | -1.4 | 2.05E-07 |
| SLC4A4 | 7.078821899 | 8.461419229 | -1.4 | 2.04E-10 |
| EDN3 | 5.336037029 | 6.722754345 | -1.4 | 8.65E-05 |
| HGD | 6.278289648 | 7.705537455 | -1.4 | 1.75E-10 |
| SEMA3D | 5.12760932 | 6.562739381 | -1.4 | 8.66E-06 |
| WNT4 | 4.485408208 | 5.927289035 | -1.4 | 5.08E-11 |
| RAG2 | 1.068310363 | 2.524360252 | -1.5 | 6.46E-07 |
| LRP1B | 3.172877341 | 4.633092102 | -1.5 | 5.17E-07 |
| C13orf36 | 3.054007915 | 4.52192567 | -1.5 | 2.57E-07 |
| SFTPC | 1.724234981 | 3.199717956 | -1.5 | 8.35E-12 |
| LOC286002 | 9.683280787 | 11.18176474 | -1.5 | 1.25E-06 |
| AGXT2L1 | 0.983718313 | 2.52678724 | -1.5 | 8.31E-13 |
| FABP4 | 4.717318203 | 6.281903811 | -1.6 | 4.22E-08 |
| MT1F | 8.625225888 | 10.20457909 | -1.6 | 7.32E-13 |
| COL9A3 | 5.530024891 | 7.112063163 | -1.6 | 7.76E-08 |
| CA4 | 4.819385549 | 6.422501313 | -1.6 | 2.39E-07 |
| FLRT1 | 3.883734269 | 5.500741168 | -1.6 | 1.38E-12 |
| DPP6 | 4.224798132 | 5.879249253 | -1.7 | 2.57E-07 |
| GATA5 | 1.977748298 | 3.639276403 | -1.7 | 1.33E-08 |
| SELV | 2.306288816 | 3.981589773 | -1.7 | 1.73E-11 |
| KIF1A | 3.319592297 | 5.005007578 | -1.7 | 6.06E-11 |
| PKHD1L1 | 5.781383282 | 7.484821231 | -1.7 | 7.43E-06 |
| STXBP5L | 3.800582781 | 5.506396975 | -1.7 | 5.07E-10 |
| TPO | 10.79531804 | 12.5063921 | -1.7 | 3.96E-07 |
| MRO | 3.631193914 | 5.351153396 | -1.7 | 4.51E-12 |
| ZNF536 | 1.924183396 | 3.657356926 | -1.7 | 5.52E-13 |
| CLCNKA | 4.127365914 | 5.911172209 | -1.8 | 2.72E-14 |
| CUX2 | 2.126759602 | 3.917783371 | -1.8 | 1.24E-08 |
| ABCC8 | 3.653777094 | 5.501772924 | -1.8 | 1.18E-09 |
| TFF3 | 6.951821585 | 8.81502019 | -1.9 | 1.37E-09 |
| WSCD2 | 4.664155921 | 6.580511961 | -1.9 | 6.42E-09 |
| ZMAT4 | 2.964894537 | 4.925612414 | -2.0 | 4.32E-09 |
| MT1H | 3.918120912 | 5.96814497 | -2.1 | 5.34E-09 |
| SLC5A8 | 4.671540539 | 6.734019672 | -2.1 | 8.96E-10 |
| DIO1 | 6.649939486 | 8.822116129 | -2.2 | 4.28E-09 |
| MT1G | 7.50526443 | 9.960797144 | -2.5 | 2.88E-12 |

Student's T-test with BH corrected p value ≤0.05; log_2_ fold change ≥ 1 or ≤ -1.

BH: Benjamini–Hochberg
