# Supplementary material for: Integrated microRNA, gene expression and transcription factors signature in papillary thyroid cancer with lymph node metastasis
Source: PeerJ. 2016 Jun 15;4:e2119. doi: 10.7717/peerj.2119 (PMC4918724; doi:10.7717/peerj.2119)
Supplement: File S2 [file peerj-04-2119-s006.docx]

#install bioconductor

source("http://bioconductor.org/biocLite.R")

biocLite()

#Read the data

Data_TC<-read.csv("combined_RNA.csv",header=T)

head(Data_TC)

colnames(Data_TC)

gene_ID<-Data_TC[,1]

Data_TC2<-Data_TC[,-1]

head(Data_TC2)

colnames(Data_TC2)

#Data Labels

colnames(Data_TC2)<-rep(c("PTC_LNM","PTC_withoutLNM","Normal"),c(205,213,59))

n1=205;

n2=213;

n3=59;

nc=477;

DataLabel<-c(rep(1,n1),rep(2,n2),rep(3,n3))

dim(Data_TC2)

colnames(Data_TC2)

rownames(Data_TC2)

rownames(Data_TC2)<-gene_ID

--------------------------------------------------------------------------

##Group data##

#PTC_LNM vs PTC_withoutLNM

colnames(Data_TC2)

PTC_LNMvsPTC_withoutLNM<-Data_TC2[,c(1:205,206:418)]

colnames(PTC_LNMvsPTC_withoutLNM)

#change format to matrix format

PTC_LNMvsPTC_withoutLNM_mat<-as.matrix(PTC_LNMvsPTC_withoutLNM)

head(PTC_LNMvsPTC_withoutLNM_mat)

#data with gene_id as a row

PTC_LNMvsPTC_withoutLNM_log<-log2(PTC_LNMvsPTC_withoutLNM_mat+1)

head(PTC_LNMvsPTC_withoutLNM_log)

dim(PTC_LNMvsPTC_withoutLNM_log)

colnames(PTC_LNMvsPTC_withoutLNM_log)

colnames(PTC_LNMvsPTC_withoutLNM_log)<-rep(c("PTC_LNM","PTC_withoutLNM"),c(205,213))

n1=205;

n2=213;

nc=418;

DataLabelPTC_LNMvsPTC_withoutLNM<-c(rep(1,n1),rep(2,n2))

## Statistical Analysis

pValue_t<-NULL

for (i2 in 1:dim(PTC_LNMvsPTC_withoutLNM_log)[1])

{

DataYY<-data.frame(YY=PTC_LNMvsPTC_withoutLNM_log[i2,],FactorLevels=factor(DataLabelPTC_LNMvsPTC_withoutLNM))

pValue_t[i2]<-t.test(YY~FactorLevels,paired=F,data=DataYY,var.equal=FALSE)[[3]]}

pValuePTC_LNMvsPTC_withoutLNM.adj<-p.adjust(pValue_t,method="BH")

sig_ttestPTC_LNMvsPTC_withoutLNM<-which(pValuePTC_LNMvsPTC_withoutLNM.adj<0.05)

length(sig_ttestPTC_LNMvsPTC_withoutLNM)

markersPTC_LNMvsPTC_withoutLNM<-PTC_LNMvsPTC_withoutLNM_log[sig_ttestPTC_LNMvsPTC_withoutLNM,]

write.csv(markersPTC_LNMvsPTC_withoutLNM,"8611_PTC_LNMvsPTC_withoutLNM.csv",row.names=T)

## Statistical Analysis and exporting p-value

pValue_t<-NULL

for (i2 in 1:dim(PTC_LNMvsPTC_withoutLNM_log)[1])

{

DataYY<-data.frame(YY=PTC_LNMvsPTC_withoutLNM_log[i2,],FactorLevels=factor(DataLabelPTC_LNMvsPTC_withoutLNM))

pValue_t[i2]<-t.test(YY~FactorLevels,paired=F,data=DataYY,var.equal=FALSE)[[3]]}

pValuePTC_LNMvsPTC_withoutLNM.adj<-p.adjust(pValue_t,method="BH")

sig_ttestPTC_LNMvsPTC_withoutLNM<-which(pValuePTC_LNMvsPTC_withoutLNM.adj<0.05)

pvaluedata<-pValuePTC_LNMvsPTC_withoutLNM.adj[sig_ttestPTC_LNMvsPTC_withoutLNM]

length(sig_ttestPTC_LNMvsPTC_withoutLNM)

rownames(sig_ttestPTC_LNMvsPTC_withoutLNM)

row_sig_pvalue<-rownames(PTC_LNMvsPTC_withoutLNM_log[sig_ttestPTC_LNMvsPTC_withoutLNM,])

sig_markers<-cbind(row_sig_pvalue,pvaluedata)

head(sig_markers)

write.csv(sig_markers,"pvalue_8611_PTC_LNMvsPTC_withoutLNM.csv",row.names=T)

-----------------------------------------------------------------------------------------------------------------------------------------

##Group data##

#PTC_LNM vs normal thyroid

colnames(Data_TC2)

PTC_LNMvsNormal<-Data_TC2[,c(1:205,419:477)]

colnames(PTC_LNMvsNormal)

#change format to matrix format

PTC_LNMvsNormal_mat<-as.matrix(PTC_LNMvsNormal)

head(PTC_LNMvsNormal_mat)

#data with gene_id as a row

PTC_LNMvsNormal_log<-log2(PTC_LNMvsNormal_mat+1)

head(PTC_LNMvsNormal_log)

dim(PTC_LNMvsNormal_log)

colnames(PTC_LNMvsNormal_log)

colnames(PTC_LNMvsNormal_log)<-rep(c("PTC_LNM","Normal"),c(205,59))

n1=205;

n2=59;

nc=264;

DataLabelPTC_LNMvsNormal<-c(rep(1,n1),rep(2,n2))

## Statistical Analysis

pValue_t<-NULL

for (i2 in 1:dim(PTC_LNMvsNormal_log)[1])

{

DataYY<-data.frame(YY=PTC_LNMvsNormal_log[i2,],FactorLevels=factor(DataLabelPTC_LNMvsNormal))

pValue_t[i2]<-t.test(YY~FactorLevels,paired=F,data=DataYY,var.equal=FALSE)[[3]]}

pValuePTC_LNMvsNormal.adj<-p.adjust(pValue_t,method="BH")

sig_ttestPTC_LNMvsNormal<-which(pValuePTC_LNMvsNormal.adj<0.05)

length(sig_ttestPTC_LNMvsNormal)

markersPTC_LNMvsNormal<-PTC_LNMvsNormal_log[sig_ttestPTC_LNMvsNormal,]

write.csv(markersPTC_LNMvsNormal,"14192_PTC_LNMvsNormal.csv",row.names=T)

## Statistical Analysis and exporting p-value

pValue_t<-NULL

for (i2 in 1:dim(PTC_LNMvsNormal_log)[1])

{

DataYY<-data.frame(YY=PTC_LNMvsNormal_log[i2,],FactorLevels=factor(DataLabelPTC_LNMvsNormal))

pValue_t[i2]<-t.test(YY~FactorLevels,paired=F,data=DataYY,var.equal=FALSE)[[3]]}

pValuePTC_LNMvsNormal.adj<-p.adjust(pValue_t,method="BH")

sig_ttestPTC_LNMvsNormal<-which(pValuePTC_LNMvsNormal.adj<0.05)

pvaluedata<-pValuePTC_LNMvsNormal.adj[sig_ttestPTC_LNMvsNormal]

length(sig_ttestPTC_LNMvsNormal)

rownames(sig_ttestPTC_LNMvsNormal)

row_sig_pvalue<-rownames(PTC_LNMvsNormal_log[sig_ttestPTC_LNMvsNormal,])

sig_markers<-cbind(row_sig_pvalue,pvaluedata)

head(sig_markers)

write.csv(sig_markers,"pvalue_14192_PTC_LNMvsNormal.csv",row.names=T)

-----------------------------------------------------------------------------------------------------------------------------------------

##Group data##

#PTC_withoutLNM vs normal thyroid

colnames(Data_TC2)

PTC_withoutLNMvsNormal<-Data_TC2[,c(206:418,419:477)]

colnames(PTC_withoutLNMvsNormal)

#change format to matrix format

PTC_withoutLNMvsNormal_mat<-as.matrix(PTC_withoutLNMvsNormal)

head(PTC_withoutLNMvsNormal_mat)

#data with gene_id as a row

PTC_withoutLNMvsNormal_log<-log2(PTC_withoutLNMvsNormal_mat+1)

head(PTC_withoutLNMvsNormal_log)

dim(PTC_withoutLNMvsNormal_log)

colnames(PTC_withoutLNMvsNormal_log)

colnames(PTC_withoutLNMvsNormal_log)<-rep(c("PTC_withoutLNM","Normal"),c(213,59))

n1=213;

n2=59;

nc=272;

DataLabelPTC_withoutLNMvsNormal<-c(rep(1,n1),rep(2,n2))

## Statistical Analysis

pValue_t<-NULL

for (i2 in 1:dim(PTC_withoutLNMvsNormal_log)[1])

{

DataYY<-data.frame(YY=PTC_withoutLNMvsNormal_log[i2,],FactorLevels=factor(DataLabelPTC_withoutLNMvsNormal))

pValue_t[i2]<-t.test(YY~FactorLevels,paired=F,data=DataYY,var.equal=FALSE)[[3]]}

pValuePTC_withoutLNMvsNormal.adj<-p.adjust(pValue_t,method="BH")

sig_ttestPTC_withoutLNMvsNormal<-which(pValuePTC_withoutLNMvsNormal.adj<0.05)

length(sig_ttestPTC_withoutLNMvsNormal)

markersPTC_withoutLNMvsNormal<-PTC_withoutLNMvsNormal_log[sig_ttestPTC_withoutLNMvsNormal,]

write.csv(markersPTC_withoutLNMvsNormal,"13392_PTC_withoutLNMvsNormal.csv",row.names=T)

## Statistical Analysis and exporting p-value

pValue_t<-NULL

for (i2 in 1:dim(PTC_withoutLNMvsNormal_log)[1])

{

DataYY<-data.frame(YY=PTC_withoutLNMvsNormal_log[i2,],FactorLevels=factor(DataLabelPTC_withoutLNMvsNormal))

pValue_t[i2]<-t.test(YY~FactorLevels,paired=F,data=DataYY,var.equal=FALSE)[[3]]}

pValuePTC_withoutLNMvsNormal.adj<-p.adjust(pValue_t,method="BH")

sig_ttestPTC_withoutLNMvsNormal<-which(pValuePTC_withoutLNMvsNormal.adj<0.05)

pvaluedata<-pValuePTC_withoutLNMvsNormal.adj[sig_ttestPTC_withoutLNMvsNormal]

length(sig_ttestPTC_withoutLNMvsNormal)

rownames(sig_ttestPTC_withoutLNMvsNormal)

row_sig_pvalue<-rownames(PTC_withoutLNMvsNormal_log[sig_ttestPTC_withoutLNMvsNormal,])

sig_markers<-cbind(row_sig_pvalue,pvaluedata)

head(sig_markers)

write.csv(sig_markers,"pvalue_13392_PTC_withoutLNMvsNormal.csv",row.names=T)
